# Optimization of *Saccharomyces cerevisiae* α-galactosidase production and application in the degradation of raffinose family oligosaccharides

**DOI:** 10.1186/s12934-019-1222-x

**Published:** 2019-10-10

**Authors:** María-Efigenia Álvarez-Cao, María-Esperanza Cerdán, María-Isabel González-Siso, Manuel Becerra

**Affiliations:** Departamento de Bioloxía, Facultade de Ciencias, Centro de Investigacións Científicas Avanzadas (CICA), Universidade da Coruña. Grupo EXPRELA, A Coruña, Spain

**Keywords:** α-Galactosidase, Biochemical characterization, Production optimization, *Saccharomyces cerevisiae*, Raffinose family oligosaccharides

## Abstract

**Background:**

α-Galactosidases are enzymes that act on galactosides present in many vegetables, mainly legumes and cereals, have growing importance with respect to our diet. For this reason, the use of their catalytic activity is of great interest in numerous biotechnological applications, especially those in the food industry directed to the degradation of oligosaccharides derived from raffinose. The aim of this work has been to optimize the recombinant production and further characterization of α-galactosidase of *Saccharomyces cerevisiae*.

**Results:**

The *MEL1* gene coding for the α-galactosidase of *S. cerevisiae* (ScAGal) was cloned and expressed in the *S. cerevisiae* strain BJ3505. Different constructions were designed to obtain the degree of purification necessary for enzymatic characterization and to improve the productive process of the enzyme. ScAGal has greater specificity for the synthetic substrate *p*-nitrophenyl-α-d-galactopyranoside than for natural substrates, followed by the natural glycosides, melibiose, raffinose and stachyose; it only acts on locust bean gum after prior treatment with β-mannosidase. Furthermore, this enzyme strongly resists proteases, and shows remarkable activation in their presence. Hydrolysis of galactose bonds linked to terminal non-reducing mannose residues of synthetic galactomannan-oligosaccharides confirms that ScAGal belongs to the first group of α-galactosidases, according to substrate specificity. Optimization of culture conditions by the statistical model of Response Surface helped to improve the productivity by up to tenfold when the concentration of the carbon source and the aeration of the culture medium was increased, and up to 20 times to extend the cultivation time to 216 h.

**Conclusions:**

ScAGal characteristics and improvement in productivity that have been achieved contribute in making ScAGal a good candidate for application in the elimination of raffinose family oligosaccharides found in many products of the food industry.

## Background

The raffinose family oligosaccharides (RFOs), consisting mainly of raffinose and stachyose, are complex sugars with one or more galactose residues joined by α-1,6-glycosidic bonds to a sucrose. These α-galactosides function as reserve polysaccharides that are stored in the vacuoles of many vegetables, especially in legumes and cereals. α-Galactosidases (α-Gals; EC 3.2.1.22) can catalyse the release of α-d-galactosyl substituents from sugars, such as melibiose, raffinose and stachyose or even polymeric galactomannans. Since monogastric animals (including man) lack pancreatic α-galactosidase (α-Gal), these indigestible carbohydrates, identified as anti-nutritional factors, can cause flatulence and other gastrointestinal disorders [1]. This fact, together with the increase in the consumption of soy products and derivatives of other legumes in human and animal food, contributes to the growing importance of the use of α-Gals in the degradation of RFOs to improve the efficiency and nutritional value of food [2] and feed [3]. Also in the sugar industry, these enzymes help us to increase the yield of sucrose after hydrolysis of raffinose that otherwise hinders the crystallization of table sugar [4]. Galactomannans or “gums” are used as additives to increase the viscosity of many foods by modifying their texture and consistency without affecting their own characteristics [5]. Depending on the degree of polymerization desired, α-Gals act synergistically with β-mannanases and β-mannosidases in hydrolysing these polysaccharides [6]. An α-Gal has even been reported that modifies the properties of gum Arabic that is widely used in food and non-food applications [7]. α-Gals can also synthesize α-GOS through transglycosylation reactions that occur under supersaturation conditions of a substrate. Some of these α-GOS can be used as therapeutic agents to prevent bacterial infections or add commercial value to products that contain them as prebiotic foods [8–10]. α-Gals are involved in many other biotechnological applications such as pulp and paper production [11], biofuels [12], blood group conversion [13] and treatment of Fabry disease [14]. This work addresses the ability of these enzymes to hydrolyse RFOs and galactomannans that could be used to improve the nutritional value of foods that are increasingly being consumed, such as soybeans and other legume derivatives.

*Saccharomyces cerevisiae* α-galactosidase ScAGal belongs to family 27 of glycosyl hydrolases (GH27) due to its similarity of amino acid sequence and structure to other α-Gals of eukaryotes [15]. It is an extracellular protein, thanks to the presence of a signal peptide that directs it to the secretory pathway [16]. Post-translational modifications produce a mature protein with 30–40% of its molecular weight in carbohydrates [17]. It has an optimum temperature of 40 °C (t_1/2_ 50 °C = 14 h, t_1/2_ 60 °C = 30 min, t_1/2_ 70 °C = 5 min), an optimum pH of 4, and > 80% of the maximal activity is retained in from pH 2 to 7.5 [18]. These are features similar to other yeast species, such as *Debaryomyces* sp. [19]. Most α-Gals isolated from eukaryotes are acidic enzymes included in the GH27 family, whereas the α-Gals identified from prokaryotes are generally neutral enzymes that belong to the GH36 family [20, 21]. The ScAGal stability at acid-neutral pH is shared with other α-Gals of mesophilic fungal origin [22–26] showing a broad range advantage for applications in the food industry. The GH families are polyspecific, i.e. many enzymes that act on a specific substrate can be found in different GH families. Another classification establishes a first group of α-Gals that are specific only for small α-galactosides, such as melibiose and RFOs, and a second group that acts on both small substrates and polymeric galactomannans [27]. However, according to their specificity on synthetic galactomannan-oligosaccharides, α-Gals can be classified into three groups depending on whether they allow the terminal, internal or both galactose to be released from previously mentioned substrates [28]. The use of this type of synthetic substrates helps to determine the exact cleavage position of the α-1,6-glycosidic bond between a D-galactosyl residue and a d-mannose residue of the linear chain of β-1,4-d-mannose of galactomannan, but the difficulty in obtaining them means that few works report such data [29–31].

There are many references to α-Gals isolated from different organisms; many of them have been identified in fungi [22, 23, 32–36], bacteria [29, 37–40], plants [41], and even in the gut of insects [42], and have been characterized because of the big industrial potential for the hydrolysis of RFOs and/or galactomannans. However, there are more current works that have informed α-Gals from original sources [22, 23, 29, 33–36, 41] than the use of expression hosts [32, 37–40, 42] for the enhanced production of enzyme. Moreover, in most cases, the enzyme purification process requires many steps, but there are few studies that have optimized overexpression α-Gals production conditions for cost-effective use on an industrial scale [43–45]. Response surface methodology (RSM) is an empirical modelling technique [46] that helps us to understand the influential variables and their interactions in biotechnological processes, since the traditional method of varying a factor each time may involve many more experiments than are really necessary. This methodology has been used previously to detect the optimum conditions for α-Gals production [43–45, 47–51]. Therefore, the aim of the present work has been to explore different secretion and purification systems for ScAGal overexpression, and efficient production optimization by RSM directed towards a potential industrial use.

## Materials and methods

### Strains, vectors and culture media

**Fig. 1:**
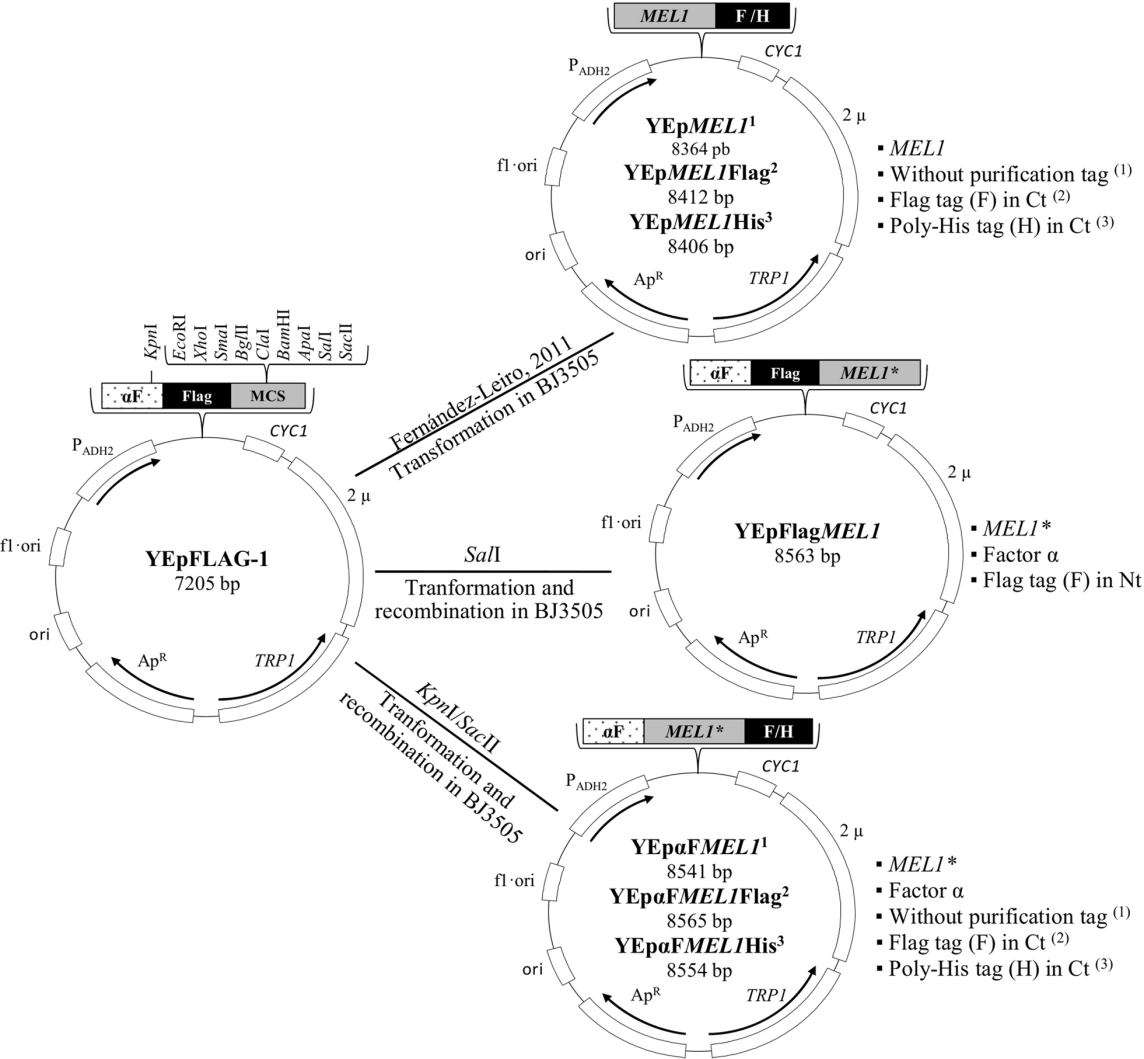
Constructs used to improve the expression of the ScAGal gene. *MEL1* *, gene *MEL1* without endogenous secretion signal; Ct, carboxyl terminal end; Nt, amino terminal end; αF, yeast secretion signal α-Factor

*Escherichia coli* XL1-Blue [*recA1 endA1 gyrA96 thi*-*1 hsdR17 supE44 relA1 lac* [*F’proAB lacIqZDM15 Tn10* (*Tetr*)]], (Stratagene Cloning Systems) was used in the propagation of plasmids by standard molecular biology techniques [52], using LB culture medium (1% (w/v) tryptone, 0.5% (w/v) yeast extract, 0.5% (w/v) NaCl), supplemented with 100 mg/L ampicillin. *S. cerevisiae* BJ3505 [*pep4::HIS3, prb*-*Δ1.6R HIS3, lys2*-*208, trp1*-*Δ101, ura 3*-*52, gal2, can1*] (Eastman Kodak Company) was the host for the expression of the ScAGal gene. LB and YPD (1% (w/v) yeast extract, 0.5% (w/v) peptone, 0.5% (w/v) glucose) media were used for the growth and maintenance of strains XL1-Blue and BJ3505, respectively. Plasmid YEpFLAG-1 [*amp*^*r*^ *ori 2μ FLAG TRP1*] (Eastman Kodak Company) and plasmid constructions YEp*MEL1*, YEp*MEL1*His and YEp*MEL1*Flag, as previously described [18], were vectors for the cloning and expression of variants of the ScAGal gene in *S. cerevisiae* (Fig. 1). As a selective culture medium, a complete medium without the amino acid tryptophan (CM-Trp) was used [53], and the modified YPHSM medium (1% (w/v) yeast extract, 8% (w/v) peptone, 1.5% (w/v) glucose, 3% (v/v) glycerol) was the high stability medium used for the expression of the heterologous protein in yeast. The culture media were supplemented with 2% (w/v) agar to make solid media. The components of the media used were sterilized by autoclaving at 121 °C for 20 min, except for ampicillin that was added later after sterilization through a 0.22 micron pore-size filter (Sartorius AG).

### Construction of gene variants of the ScAGal

The *MEL1* gene (GenBank accession no. X03102) coding for ScAGal (UniProt P04824) was amplified by PCR without the endogenous secretion signal from the vector YEp*MEL1*His [*amp*^*r*^ *ori 2μ MEL1His TRP1*]. Purification tags (Poly-His or Flag peptides) are coded by sequences of nucleotides, which were added to primers to create different ScAGal variants. In the PCR reaction, 50 ng template DNA, 1.55 min copy time, 57 °C annealing and the rest of conditions, as specified by the manufacturer of the high-fidelity DNA polymerase Vent DNA polymerase (New England Biolabs), were used. Amplified and purified PCR products (GeneJEt Gel Extraction Kit, Thermo Fisher Scientific) were cloned in the vector YEpFLAG-1, previously linearized with *Sal*I or *Kpn*I and *Sac*II, by homologous recombination in competent BJ3505 cells transformed by the lithium acetate method [54] (Fig. 1). The transformant colonies were selected in CM-Trp by complementation of auxotrophy, and identification of recombinants was relied on PCR analysis of the transformant colonies, using the Taq polymerase (DreamTaq polymerase, Thermo Fisher Scientific). Finally, after propagation and purification (GeneJET Plasmid Miniprep Kit, Thermo Fisher Scientific) of the selected candidates, each of the variants of the gene of interest was verified by sequencing (Servizos de Apoio á Investigación, Universidade da Coruña). Oligonucleotides used as primers in the amplification and sequencing reaction are shown in Additional file 1: Table S1.

### Heterologous expression of gene variants of the ScAGal

BJ3505 cells transformed with the plasmids YEp*MEL1*, YEp*MEL1*His, YEp*MEL1*Flag, YEpFlag*MEL1*, YEpαF*MEL1*, YEpαF*MEL1*His and YEpαF*MEL1*Flag, were seeded in CM-Trp plates and incubated at 30 °C for 48–72 h. In each case, a single colony was selected to prepare a pre-culture in CM-Trp until stationary phase (30 °C, 250 rpm, 72 h), which was used as an inoculum of YPHSM media until it reached an OD_600_ of 0.5. Cultures were carried out in triplicate in Erlenmeyer flasks with 20% volume of medium at 250 rpm and 30 °C.

Samples were taken at regular time-intervals to determine cell growth, enzymatic activity and plasmid stability. Plasmid stability was determined by seeding diluted samples of YPHSM cultures on CM and CM-Trp plates, and counting the isolated colonies [% plasmid stability = (Colony Forming Units _CM-Trp_/Colony Forming Units _CM_) × 100]. A simple analysis of variance (ANOVA) was used to determine statistically significant differences between the means of the designed expression systems (StatGraphics Plus version 5.1).

### Purification of ScAGal

Strain BJ3505 transformed with YEp*MEL1*His was grown for 96 h in 1 L YPHSM before being centrifuged (7000 rpm for 10 min, 4 °C), and the supernatant was filtered through 0.45 µm nitrocellulose membrane filters (Millipore) before being dialysed and concentrated by tangential filtration (TFF, Millipore) with Na_2_HPO_4_/NaH_2_PO_4_ 100 mM, (pH 7) to 100 mL. The concentrated sample was purified by affinity chromatography on a 5 mL nickel-Sepharose column (HisTrap FF Crude, GE Healthcare) coupled to the ÄKTA prime plus system (GE Healthcare), which was equilibrated with 100 mM PBS (pH 7), 500 mM NaCl, and 25 mM imidazole. After loading the sample, it was washed with 10 column volumes of the same buffer. The protein of interest was eluted with the same buffer containing 500 mM imidazole, and 1 mL fractions were collected through the system collector. When deglycosylated protein was required, after digestion with Endo H (New England Biolabs), a second purification step was included by gel filtration chromatography, using Hi-Load Superdex 200 16/60 column (GE Healthcare) pre-equilibrated with 20 mM Tris–HCl (pH 7.4) and 150 mM NaCl. Fractions with α-galactosidase activity were pooled, concentrated and dialyzed as necessary in the same buffer, using 30 kDa ultrafiltration membranes (Amicon Ultra, Millipore). Finally, the pure protein was frozen in liquid nitrogen and lyophilized for long-term preservation (freezing conditions: − 40 °C, 0.010 mBar; sublimation conditions: 25 °C, 0.010 mBar, 24 h; Telstar’s LypoQuest). The sizes of the different molecular states and the purity of the sample were determined by SDS-PAGE, Native-PAGE and Coomassie staining [55].

### Enzyme activity assay and protein concentration determination

Routine assays of α-galactosidase activity were carried out by a modified method of Ryan et al. [56]. A volume of the intracellular or extracellular diluted enzyme preparation was incubated at 40 °C with a volume of 10 mM *p*-nitrophenyl-α-d-galactopyranoside (PNPG, Sigma Aldrich) in McIlvaine buffer (61 mM citric acid and 77 mM Na_2_HPO_4_, pH 4). After stopping the reaction at different time-intervals with a volume of 1 M Na_2_CO_3_, the *p*-nitrophenol released was measured at 400 nm (molar extinction coefficient, 18.20 L/mmol/cm). One activity unit (U) was defined as the amount of enzyme that releases one μmol of *p*-nitrophenol per minute under assay conditions. Protein concentration was determined by the Bradford method using a DC Protein Assay Kit (Bio Rad) and bovine serum albumin as standard of the calibration line. Samples were taken in all cases in triplicate, and the spectrophotometric readings were made in flat-bottom microtiter plates with Synergy H1 Hybrid Multi-Mode Reader (BioTEk).

### Stability assay under refrigeration and freezing conditions

Different batches of pure and partially purified protein were used to study enzymatic stability at room temperature (22 ± 2 °C), 4 °C and -20 °C by analysing the residual activity according to the standard method (“Enzyme activity assay and protein concentration determination” section).

### Treatments with proteases

Protease resistance was assayed by incubating a purified ScAGal sample (1 U/mL) at 37 °C with different acid-neutral proteases (2 mg/mL) at a ratio of 1:1 (v/v) according to the specifications of the manufacturer: pepsin in citrate–phosphate buffer (pH 2.5) and proteinase K, trypsin, α-chymotrypsin type II and subtilisin in 0.1 M Tris–HCl (pH 7.4). The protease resistance of a previously deglycosylated ScAGal sample, as described above (“Purification of ScAGal” section), was also tested. As a positive control of proteolysis, the β-galactosidase enzyme of *E. coli* was used under the same reaction conditions, and its enzymatic activity was determined qualitatively and quantitatively using the chromogenic substrates X-gal (5-Bromo-4-chloro-3-indolyl-β-d-galactopyranoside) and ONPG (2-nitrophenyl-β-d-galactopyranoside), respectively. Samples were taken at different reaction times and residual enzymatic activity was determined by standard methods (“Enzyme activity assay and protein concentration determination” section) using as control sample the enzyme in the absence of protease taken as 100% activity. Quantitative assay of β-galactosidase activity followed the manufacturer’s specifications, and later, 1 μL X-gal (20 mg/mL in dimethylformamide), was added (qualitative assay). Proteases, substrates and β-galactosidase from *E. coli* were supplied by Sigma Aldrich.

### Substrate specificity determination

For ScAGal substrate specificity characterization, substrates PNPG, melibiose, raffinose, stachyose and locust bean galactomannan from Sigma Aldrich, and substrates Gal^1^Man_3_ and Gal^3,4^Man_5_ together with *Helix pomatia* β-mannosidase acquired from Megazyme, were used. The hydrolysis products were analysed by thin layer chromatography (TLC) and high performance liquid chromatography (HPLC). The α-Gals of *Aspergillus niger* and *Cyamopsis tetragonoloba* from Megazyme were used for a comparative study of the hydrolysis products. Each reaction and the corresponding controls were prepared in triplicate.

#### Determination of α-galactosidase activity

Enzyme specificity by the synthetic substrate PNPG was determined by measuring the release of *p*-nitrophenol at 400 nm (“Enzyme activity assay and protein concentration determination” section). The specificity for raffinose, stachyose and locust bean galactomannan was determined by measuring the release of reducing sugars, using the DNS reagent [57]. In the samples of locust bean galactomannan previously digested with β-mannosidase, the amount of galactose liberated was quantified by the difference between the reducing sugars released before and after the treatment of the samples with ScAGal. The GOD-POD kit (Sigma Aldrich) was used to determine the enzymatic activity in the presence of melibiose by measuring the release of glucose. One unit of activity (U) was defined as the amount of enzyme releasing 1 μmol product (galactose or glucose) per minute under the assay conditions.

#### Enzymatic hydrolysis

The hydrolysis reaction [58] was carried out in each case, at 40 °C with a mixture (1:1, v/v) of an aqueous solution of 5 mg/mL of each substrate and 1 U/mL α-Gals tested (in reaction buffer). On the other hand, to determine the breaking position of bonds between galactose and mannose, and the hydrolysis efficiency on complex galactomannan-oligosaccharides, 1.2 U/mL β-mannosidase in 5 mM acetate buffer (pH 4.5) was incubated at 40 °C for 24 h with a 1% (w/v) aqueous solution of synthetic galactomannan-oligosaccharides (Gal^1^Man_3_ and Gal^3,4^Man_5_) and locust bean galactomannan, respectively (1:1, v/v). After stopping the reaction, part of the previous hydrolysate was incubated with 0.3 U/mL ScAGal (in reaction buffer) at 40 °C for 24 h (1:1, v/v). Samples collected were heated at 100 °C for 5 min to stop the reaction before being analysed. McIlvaine buffer was used as a reaction buffer for ScAGal, and α-Gal from *A. niger*, whereas 10 mM acetate buffer (pH 4.5) was used for α-Gal from *C. tetragonoloba*.

#### Analysis of hydrolysis products by TLC and HPLC

In the TLC analyses, each sample was placed with a capillary on silica-gel plates (Merck Silica Gel 60F 254, Germany) and the hydrolysis products were developed on the solvent system 1-propanol-nitromethane-water (5:2:3, v/v). Sugars were detected by heating in an oven after spraying the plates with a mixture of methanol:sulfuric acid (95:5, v/v). Images and analyses of the plates were collected with a transilluminator (Molecular Imager Gel Doc XR+, BioRad). In the HPLC analysis, Sugar Pack Waters column (6.5 mm × 300 mm) and 100 μM EDTA-Calcium (Sigma Aldrich) were used as the mobile phase (column temperature, 80 °C; sensor temperature, 37 °C; sensitivity, 32; flow, 0.5 mL/min). Sugars that were eluted were detected with a Refractive Index Detector (Cienytech). The identification and quantification of sugars by HPLC involved a method using sorbitol (1 mg/mL) as internal standard [59]. A mixture of melibiose, glucose and galactose was used as external standard for the hydrolysis of melibiose, whereas a mixture of stachyose, raffinose, sucrose and galactose was used for the rest of substrates. The calibrated lines were performed using aqueous solutions between 4 and 0.06 mg/mL of each external standard.

### Statistical response surface model

RSM was the statistical method used to improve the production of ScAGal by the recombinant strain BJ3505/YEp*MEL1*His in the YPHSM medium. The parameters selected as experimental factors were aeration, glucose concentration, pH and time of growth; the measured response was the extracellular α-galactosidase activity of the cultures. pH was adjusted by adding 1 M NaOH or 1 M HCl, and the rest of culture conditions were set according to the procedure described in “Heterologous expression of gene variants of the ScAGal” section. A central composite design (CCD) was used to study the effects of the factors (as independent variables) on the response (dependent variable) at 5 different levels. The coded values of the levels were, − α, − 1, 0, + 1, + 2, + α, where α = 2^k/4^, k is the number of independent variables and 0 corresponds to the central point of the experimental domain. The values of the factors for the central point were chosen after a series of preliminary experiments, and the correspondence between the coded and real values of the independent variables is shown in Table 1. To estimate the experimental error, the central point (all factors at zero level) was repeated 6 times. The measured response was adjusted to the independent variables using a second-order polynomial equation. Statistical data were analysed with the help of StatGraphics Plus for Windows version 5.1 (Statistical Graphics Corporation).

**Table 1:** Experimental factors and CCD levels in the optimization of the production of ScAGal by BJ3505/YEp*MEL1*His

Real values	Coded values^a^				
	− 2	− 1	0	1	2
Aeration; A (Vc/Ve)^b^	0.5 (50/100)	0.6 (40/100)	0.7 (30/100)	0.8 (20/100)	0.9 (10/100)
Glucose (%); G	0.5	1.0	1.5	2.0	2.5
pH; P	4	5	6	7	8
Time (h); T	48	72	96	120	144

^a^*x*_*i*_= (X_*i*_−X_*0*_)/∆X_*i*_; where *x*_*i*_ and X_*i*_ are the coded and real values of the independent variable *i*, X_0_ is the real value of the independent variable *i* at the central point, and ∆X_*i*_ is the step change value. ^b^Aeration = 1 − (Vc/Ve); volume ratio of culture medium in mL (Vc) in a 100 mL Erlenmeyer flask (Ve)

## Results and discussion

### Monitoring the expression of variants of MEL1

**Fig. 2:**
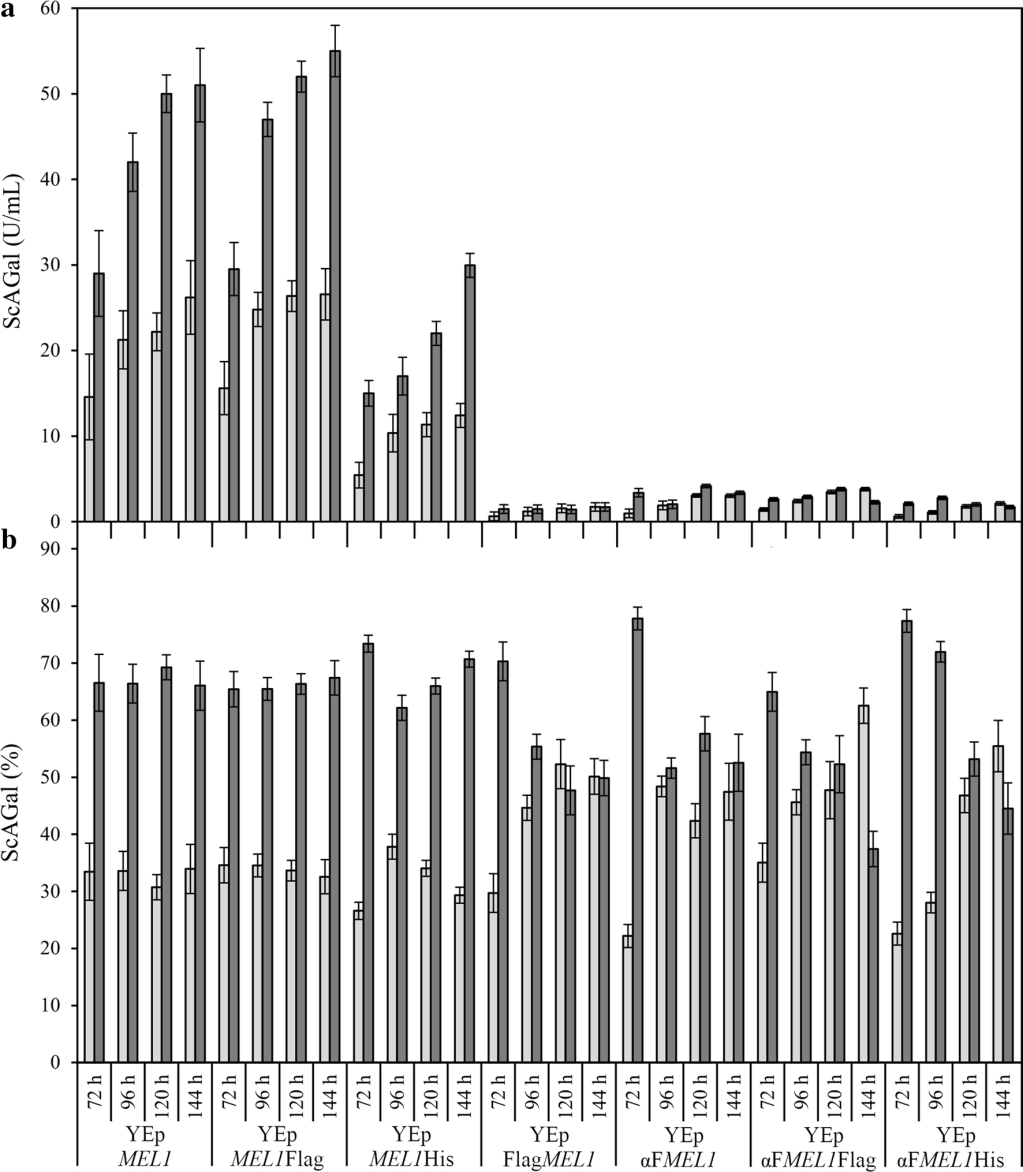
Monitoring of variants *MEL1* expressed in strain BJ3505 using YPHSM culture medium. Extracellular (clear grey), intracellular (dark grey) activity (**a**), and percentage of activity of the extracellular (clear grey) and intracellular (dark grey) fraction, with respect to total activity (**b**). YEp*MEL1*, YEp*MEL1*Flag and YEp*MEL1*His contain the full *MEL1* gene, while in YEpFlag*MEL1*, YEpαF*MEL1*, YEpαF*MEL1*Flag and YEpαF*MEL1*His the native secretion signal of ScAGAL was replaced by the signal α-Factor. Mean ± DS, N = 3

To select the best secretion and purification system for the ScAGal protein, different variants of the *MEL1* gene were expressed in strain BJ3505 using YPHSM medium. The constructions previously described, YEp*MEL1*, YEp*MEL1*His and YEp*MEL1*Flag [18], carrying the *MEL1* gene with its native secretion signal, were used. In addition, other constructions were generated (YEpFlag*MEL1*, YEpαF*MEL1*, YEpαF*MEL1*His and YEpαF*MEL1*Flag) with the absence or presence of purification tags, and, in which, by homologous recombination, the native secretion signal was replaced by the α-factor signal of *S. cerevisiae* provided by YEpFLAG-1 (Fig. 1). The signal peptide is present at the amino-terminal of the newly synthesized protein, directs it towards the secretory pathway, and is cleaved by specific proteases before the protein is released into the extracellular medium. Although signal peptides are extremely heterogeneous, and many can be functionally interchangeable between different species [60], the efficiency of protein secretion is strongly determined by them [61, 62]. Since the *ADH2* promoter in the plasmid is subject to catabolic repression by glucose: YPHSM medium using 1.5% (w/v) glucose instead of 1% (w/v) permits an increasing number of cells prior to induction of gene expression (previously observed data). Figure 2 shows the course of the intra- and extra-cellular α-galactosidase activity of the cultures with time. Although the cultures in all cases reached the stationary phase at 96 h with similar growth values (OD_600_ = 90 ± 10, not shown), the presence of the native signal peptide led to the recombinant strain secreting an average activity ~ 10 times higher than with the α-factor. The best expression was obtained with the constructions YEp*MEL1*Flag, YEp*MEL1* and YEp*MEL1*His, reaching an extracellular activity at 96 h of 25, 21 and 10 U/mL, respectively (Fig. 2a). ANOVA shows that the highest activity was in the BJ3505/YEp*MEL1*Flag system (group B, extracellular α-galactosidase activity data obtained with the strain BJ3505/YEp*MEL1*Flag), followed by BJ3505/YEp*MEL*1 (group A, extracellular α-galactosidase activity data obtained with the strain BJ3505/YEp*MEL*1) and BJ3505/YEp*MEL1*His (group C, extracellular α-galactosidase activity data obtained with the strain BJ3505/YEp*MEL1*His). Therefore, the statistical bias and standardized kurtosis are both within the expected range for data that is normally distributed (− 2 and + 2). Thus, the F-ratio showed that there were statistically significant differences between groups A, B and C (p-value < 0.05), and finally, groups A and B were not significantly different on a multiple-range test (Additional file 2: Table S2).

Whereas the average yield of ScAGal secretion directed by the α-factor was 45–50% with respect to the total activity at 120 h, secretion directed by the native signal was 30% over the same time interval (Fig. 2b). That intracellular activity is greater than the extracellular activity may be due to the fact that production of ScAGal is too fast to be processed efficiently by the secretory route. Besides, the α-galactosidase activity associated with the cell wall of the yeast could be an attractive alternative in increasing the added value of the biomass generated for its application as a food supplement. YEp*MEL1*Flag contains the Flag tag in its carboxy-terminal (C-terminal) for the protein specific detection by immunoaffinity. In our previous works, YEp*MEL1*Flag helped us to obtain highly pure ScAGal necessary for crystallographic resolution [63]; however, the purification stage is too expensive to drive a large-scale production process. YEp*MEL1*, since it does not contain a purification tag, makes it more difficult to obtain the pure protein. YEp*MEL1*His expresses a fusion protein with a Poly-His tag (6 Histidines) at the C-terminal for affinity purification. Therefore, we consider BJ3505/YEp*MEL1*His as the best ScAGal expression system since it offers a cheap and simple purification method that yields a higher profit margin over production cost.

Finally, the monitoring of cultures in YPHSM medium with time shows a plasmid stability of up to 80% during the 96 h of growth, decreasing up to 30% as the culture time extends to 216 h (Additional file 3: Fig. S1A). This is a higher plasmid stability than reported for other proteins [64]. Plasmid loss is expected because the recombinant strain in YPHSM is not subjected to selective pressure, whereas the using CM-Trp as a selective medium limits cell density and enzyme production. In this sense, BJ3505/YEp*MEL1*His growing in CM-Trp secretes an average of 0.53 U/mL between 96 and 140 h [18], whereas it reaches an average of 11 U/mL over the same time interval when it grows in YPHSM (Additional file 3: Fig. S1B).

### Purification of the ScAGal

Cell-free BJ3505/YEp*MEL1*His culture medium was concentrated and partially purified by ultrafiltration. The extracellular protein was then purified in a single step by affinity chromatography on nickel-Sepharose column (as described in “Purification of ScAGal” section). Table 2 summarizes the purification protocol, showing that recovery of the protein by 1.14% with a tenfold purification factor and an activity yield of 13%. During the dialysis, concentration and lyophilisation stages, a loss of 20% of purified protein was assumed. However, it should be noted that, although in this case the extracellular production of ScAGal resulted in a specific activity of 12.61 U/mg (Table 2), this could be improved during the optimization process of the culture of the recombinant strain (“Optimization of ScAGal production by RSM” section) to reach further increase in expression levels.

**Table 2:** Summary of the purification of ScAGal

Purification step		U^a^	mg	U/mg	PF^b^	Yield	
						(U, %)	(mg, %)
Extracellular medium	1000 mL	3530	280	12.61	1.0	100	–
Concentrated medium	100 mL	2598	160	16.23	1.3	74	–
Affinity chromatography	10 mL	454	3.5	129.60	10.3	13	100
Dialysis + concentration	1 mL	238	3.2	74.55	–	7	91
Lyophilisation^c^	169 mg	207	2.8	73.85	–	6	80

^a^Extracellular α-galactosidase activity was determined using PNPG as substrate (40 °C, pH 4). ^b^Purification factor. ^c^mg dry weight

SDS-PAGE analysis of each of the purification steps allowed us to check the state and final purity of the protein (Additional file 4: Fig. S2A). A diffuse band between 180 and 70 kDa was observed, which confirms the high glycosylation state that represents 50% of the final molecular weight (MW) of the protein (Additional file 4: Fig. S2B, lane 1) as previously reported [65]. Besides, deglycosylated protein has a MW of 55 kDa (Additional file 4: Fig. S2B, lanes 2, 3 and 4) and maintains ~ 100% of its initial activity [65], an important quality depending on its industrial application. The deglycosylation of α-Gal of *C. arietinum* reduces enzymatic activity [13]. A native gel of the pure protein, before and after deglycosylation (Additional file 4: Fig. S2C, lanes 1 and 2, respectively), shows its oligomeric nature corresponding to the tetrameric state of ScAGal found in the crystal structure [65].

### Conservation conditions of the ScAGal

In order to assess the best method of conserving the enzyme, a stability study was carried out in cold and environmental conditions (Additional file 5: Fig. S3). Aqueous samples of ScAGal purified by affinity chromatography (136 U/mg) conserved for 5 years at 4 °C and − 20 °C maintained a residual activity of 68% and 93%, respectively. Furthermore, the enzyme retained 80% activity after 22 days at room temperature (RT; 22 °C ± 2), although longer times have not been tested due to evaporation problems. On the other hand, partially purified ScAGal by tangential filtration (16 U/mg) had > 80% residual activity after 5 years at − 20 °C. Long-term maintenance at RT or refrigeration conditions is not recommended due to the high risk of contamination if the protein has not been fully purified (data not shown). We recommend lyophilisation of the protein to keep ScAGal for long periods of time at RT.

### Resistance to proteases

**Fig. 3:**
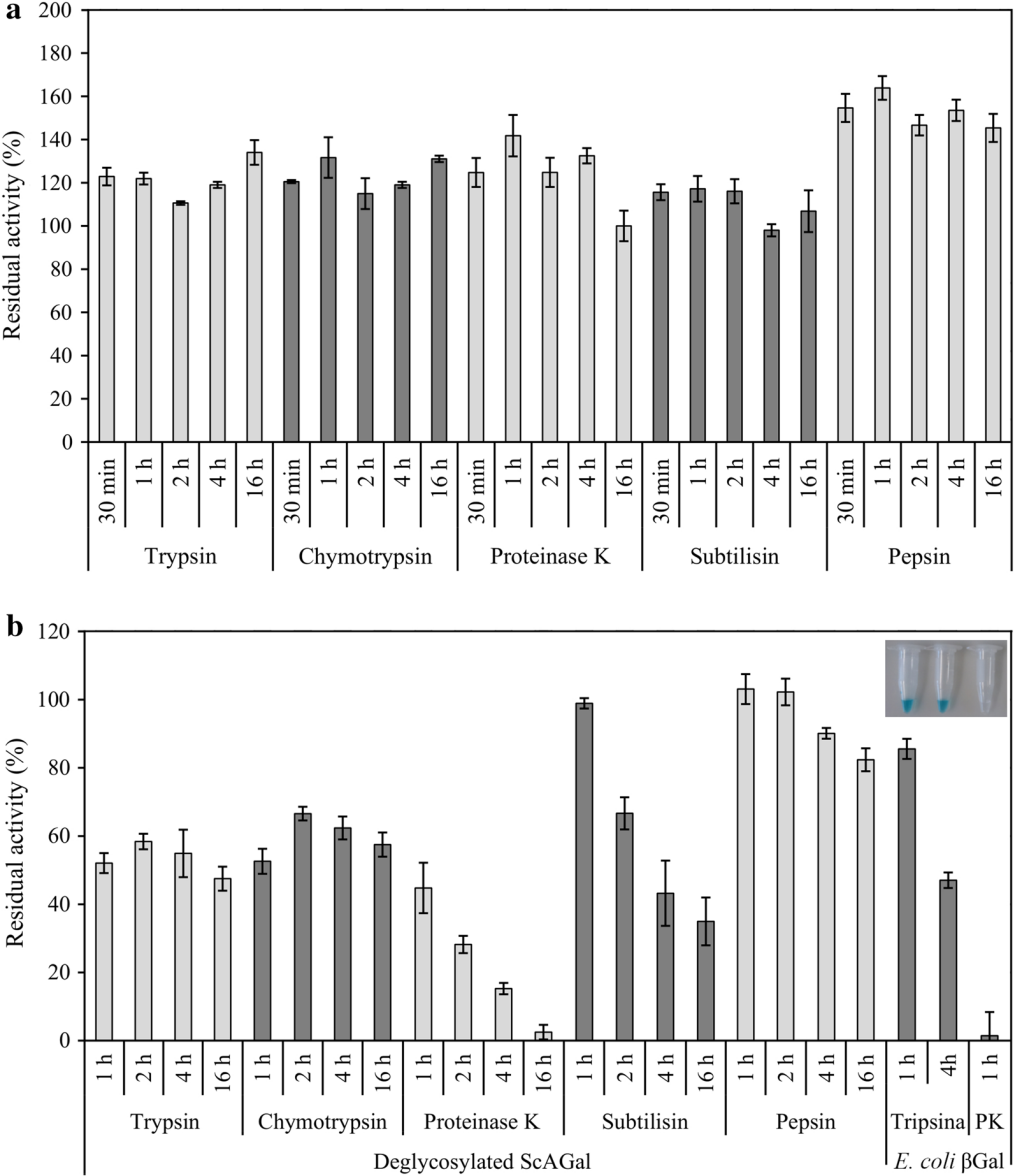
Effect of acid-neutral proteases on the activity of ScAGal. Each protease (2 mg/mL) dissolved in its reaction buffer was incubated at 37 °C in 1 U/mL ScAGal (**a**) and deglycosylated ScAGal (**b**), and the activity was measured at different times of digestion. The negative control was the enzyme acting in the absence of protease, and positive control was β-galactosidase *E. coli* (βGal) under the same assay conditions. Insert: qualitative determination of β-galactosidase activity using the chromogenic substrate X-gal (hydrolyzed product was blue). Mean ± DS, N = 3

ScAGal not only has strong resistance to treatment with all the proteases tested, but also a higher enzymatic activity in their presence (Fig. 3a). After 37 °C for 1 h with trypsin, chymotrypsin, proteinase K, subtilisin and pepsin, the enzyme’s residual activity was 122, 132, 142, 117 and 164%, respectively, varying slightly up to 16 h. The protease resistance of several α-Gals has been tested, but it was noted that most trials used short incubation times of 30–60 min, without longer incubation times being tested. ScAGal was more protease-resistance compared to most others. The enzymes PCGI from *Pleurotus citrinopileatus* [23] and Aga-BC7050 [38] were activated with α-chymotrypsin and trypsin, but were inhibited by proteinase K. ABGI from *Agaricus bisporus* only showed resistance to α-chymotrypsin [34]. TtGal27A from *Thielavia terrestris* [32], PDGI from *Pleurotus djamor* [35] and rAgas2 isolated from the gut metagenome of *Hermetia illucens* [42] retained 90, 60 and 70%, respectively, of the initial activity in the presence of neutral proteases. On the other hand, the deglycosylated ScAGal was also slightly activated by pepsin (103% residual activity) and showed strong resistance to subtilisin (98% residual activity), and some tolerance to trypsin (52% residual activity), chymotrypsin (53% residual activity) and proteinase K (45% residual activity) after 1 h treatment (Fig. 3b). In the food and feed industry, combinations of enzymes are used, including α-Gals, β-mannanases, β-mannosidases and proteases, which must work synergistically to improve the nutritional value and digestibility of food [3]. These enzymes have to resist the acidic pH of the gastric juice where the pepsin acts and later, on reaching the small intestine, they must be resistant to trypsin and chymotrypsin secreted by the pancreas, together with bicarbonate to neutralize the pH. Therefore, proteases resistance is a useful characteristic in expanding the field of action of ScAGal in biotechnological applications.

### Characterization of substrate specificity

#### α-Galactosidase activity

Purified ScAGal was used to determine substrate specificity with PNPG, melibiose, raffinose, stachyose and locust bean galactomannan (Additional file 6: Table S3). Like the great majority of α-Gals, the enzyme shows greater affinity for the synthetic PNPG substrate than for natural substrates, as previously determined [65]. Taking the PNPG (100%) as a reference, the relative activity data show that the enzyme is more specific for melibiose (95%), followed by raffinose (15%) and stachyose (11%), which corresponds to the rate of hydrolysis detected by HPLC and TLC, as will be mentioned later. ScAGal does not act directly on complex galactomannans, such as locust bean gum, but can act when complex galactomannans have been treated with β-mannosidase. This is advantageous in the application of this enzyme combinated with β-mannosidases and/or β-mannanases to improve the gelling properties of galactomannans used in the biotechnology industry [3].

**Fig. 4:**
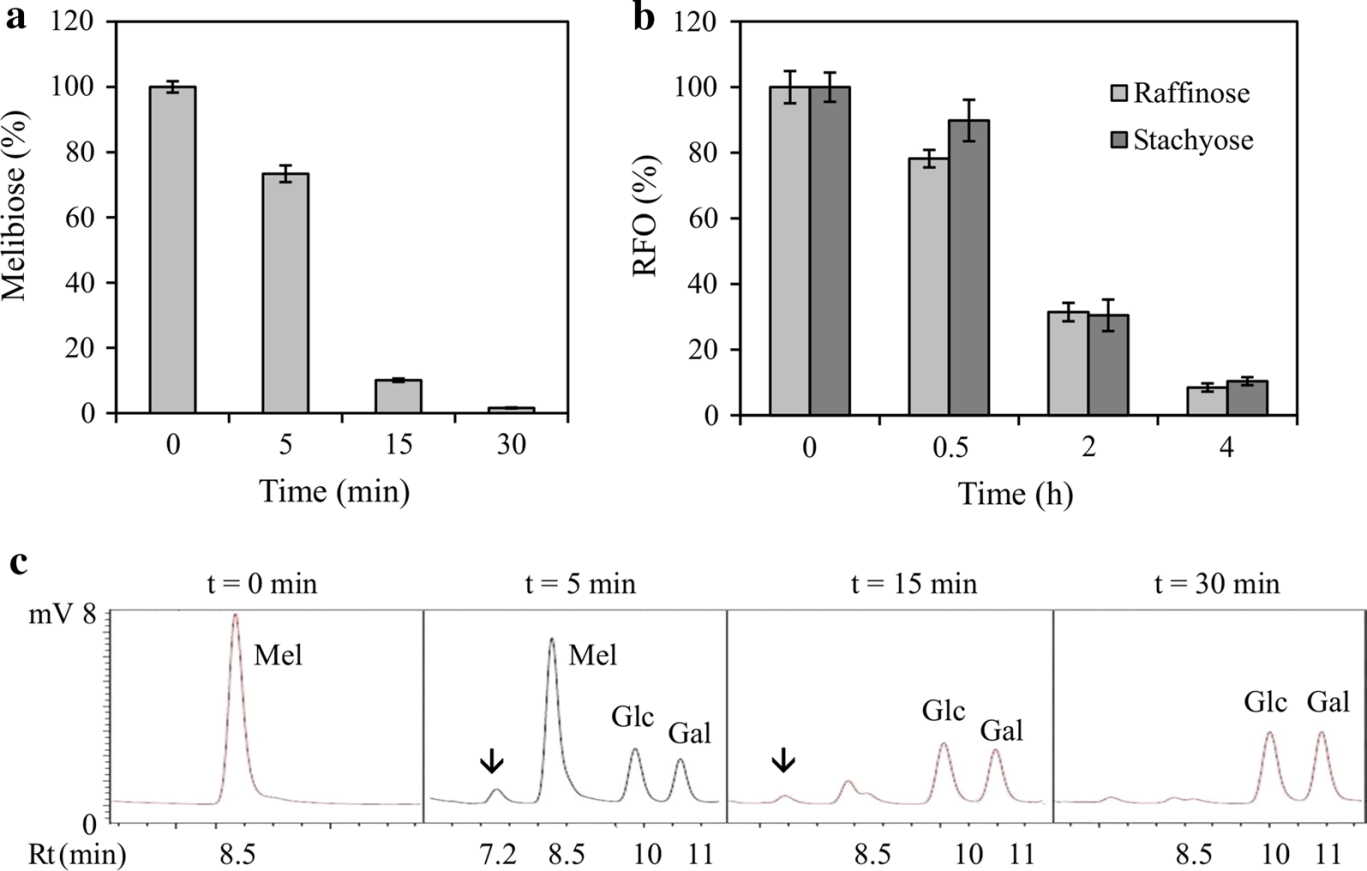
HPLC analysis of melibiose, raffinose and stachyose hydrolyzed by ScAGal. Percentage of the content of melibiose (**a**) and RFOs (**b**), and identification of melibiose hydrolysis products (**c**), during the reaction time (t); Mean ± DS, N = 3. → Tri-galactosaccharide synthesized by transglycosylation by ScAGal; mV, millivolts; Rt, retention time; Mel, melibiosa; Glc, glucose; Gal, galactose

*Analysis of hydrolysis products.* HPLC analysis showed that ~ 90% of the melibiose and RFOs (raffinose, stachyose) are hydrolysed by ScAGal at 15 min and 4 h reaction, respectively (Fig. 4a, b). Enzyme displayed higher affinity for melibiose (K_cat_ = 193/S, k_cat_/K_m_ = 17/S/mM) than raffinose (K_cat_ = 46.8/S, k_cat_/K_m_ = 0.9/S/mM) [65]. At the start of the hydrolysis reaction (5 min), the synthesis of a galactotrisaccharide as a product of the transglycosylation reaction takes place simultaneously (Fig. 4c). This happens because there is enough substrate to favour the synthesis reaction, but as time goes passes, both melibiose and galactotrisaccharide disappear. This result confirms that ScAGal can carry out transglycosylation reactions if we favour melibiose supersaturation conditions, suggesting a possible application in the synthesis of α-GOS. ScAGal can hydrolyse Gal^3^Man_3_ but cannot act on Gal^3^Man_4_ [66], which means that it can break the bond between a galactose and mannose residue from the non-reducing end of the galactomannan-oligosaccharide, but not the binding to an internal mannose. However, there is no documented evidence of its action on a galactose attached to the mannose at the reducing end of this type of substrate, such as Gal^1^Man_3_.

**Fig. 5:**
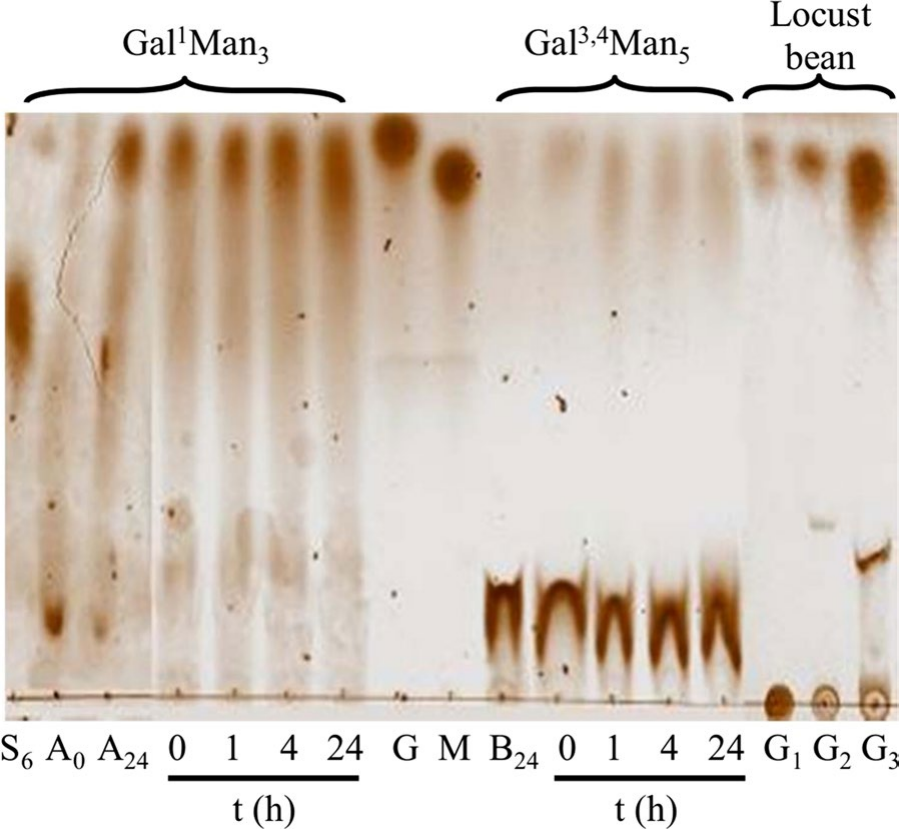
TLC analysis of the hydrolysis products of Gal^1^Man_3_, Gal^3.4^Man_5_ and locust bean gum by ScAGal. 0.3 U/mL ScAGal were incubated at 40 °C with 2.5 mg/mL of the substrate previously hydrolysed with 1.2 mL β-mannosidase, and samples were collected at 1, 4 and 24 h of reaction. Gal^1^Man_3_ (A_24_), Gal^3,4^Man_5_ (B_24_) and locust bean gum (G_2_) were digested with β-mannosidase for 24 h. S_6_, Gal^1^Man_3_ control; A_0_ shows Gal^1^Man_3_ at zero reaction time; G, galactose control; M, mannose control; G1, galactomannan control; G_3_ shows G_2_ hydrolysed with ScAGal for 24 h.; 8 μL of sample and 4 μL of control were used

**Fig. 6:**
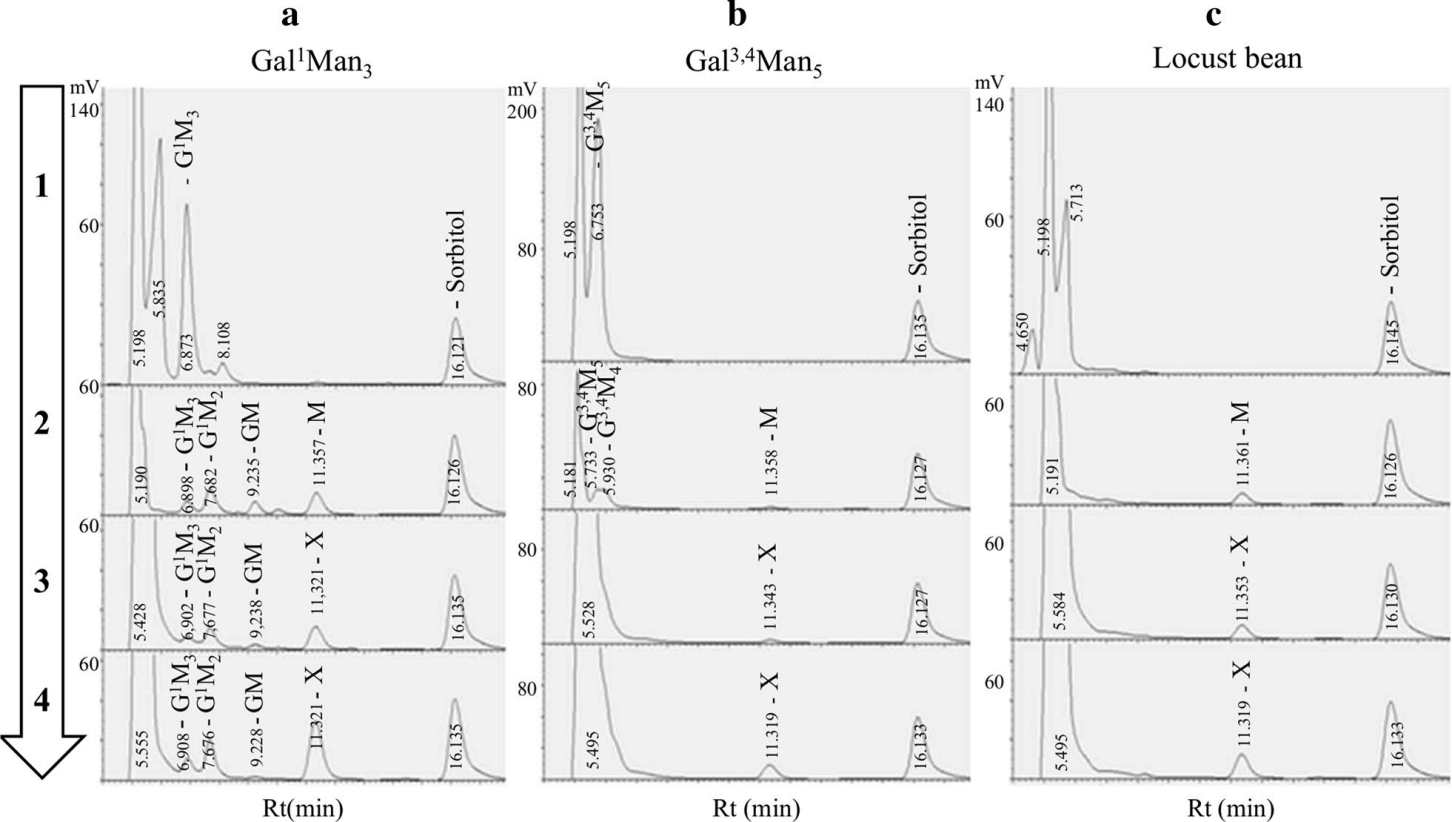
HPLC analysis of Gal^1^Man_3_ (**a**), Gal^3.4^Man_5_ (**b**) and locust bean gum (**c**) hydrolysed by ScAGal. Identification of the products obtained after digestion with β-mannosidase at 0 and 24 h of reaction (1 and 2), and after the action of ScAGal (0.3 U/ml in McIlvaine buffer, pH 4) for 4 and 24 h of the samples previously treated with β-mannosidase (3 and 4). G^1^M_3_, Gal^1^Man_3_; G^1^M_2_, Gal^1^Man_2_; GM, Gal^1^Man_1_; G^3.4^M_5_, Gal^3.4^Man_5_; G^3.4^M_4_, Gal^3.4^Man_4_; M, Mannose; X, Mannose and Galactose; mV, millivolts; Rt, retention time

The result of the action of ScAGal on Gal^1^Man_3_, Gal^3,4^Man_5_ and locust bean gum previously treated with β-mannosidase have been analysed by TLC (Fig. 5) and HPLC (Fig. 6). Gal^1^Man_3_ led to release of the terminal non-reducing mannose generating the product Gal^1^Man_2_, and finally, GalMan after 24 h, which could be accessible by ScAGal releasing galactose and mannose (Figs. 5, 6a). TLC analysis of Gal^3,4^Man_5_ hydrolysis is more confusing, but the data were finally confirmed by HPLC. Since β-mannosidase removes mannose from the non-reducing end of the linear chain of β-1,4-mano-oligosaccharide until reaching a galactose residue, its action on galactomannans, such as in locust bean, exposes galactose groups at the non-reducing end, which are rapidly hydrolysed by ScAGal (Figs. 5, 6b, c). Although the HPLC-RID chromatographic system does not allow one to separate mannose from galactose, an increase in the concentration of the product occurs after the action of the ScAGal with the same retention time (Rt), indicating release of galactose. Uncharacterized peaks were identified with the Rt of the reaction buffer components (data not shown). Depending on the specificity on synthetic galactomannan-oligosaccharides, α-Gals that release galactose that attacks the non-reducing mannose, but at the same time do not release the internal galactose residues, are classified in a first group; those that only release residues attached to internal positions constitute a second group; and those that release both internal and terminal residues are a third group [28]. Therefore, we have demonstrated that ScAGal only acts on Gal^1^Man_3_ and Gal^3,4^Man_5_ previously hydrolyzed with β-mannosidase (eliminating terminal galactose from the non-reducing end), and thus confirm that it belongs to the first group of α-Gals, according to galactomannan-oligosaccharide specificity.

**Fig. 7:**
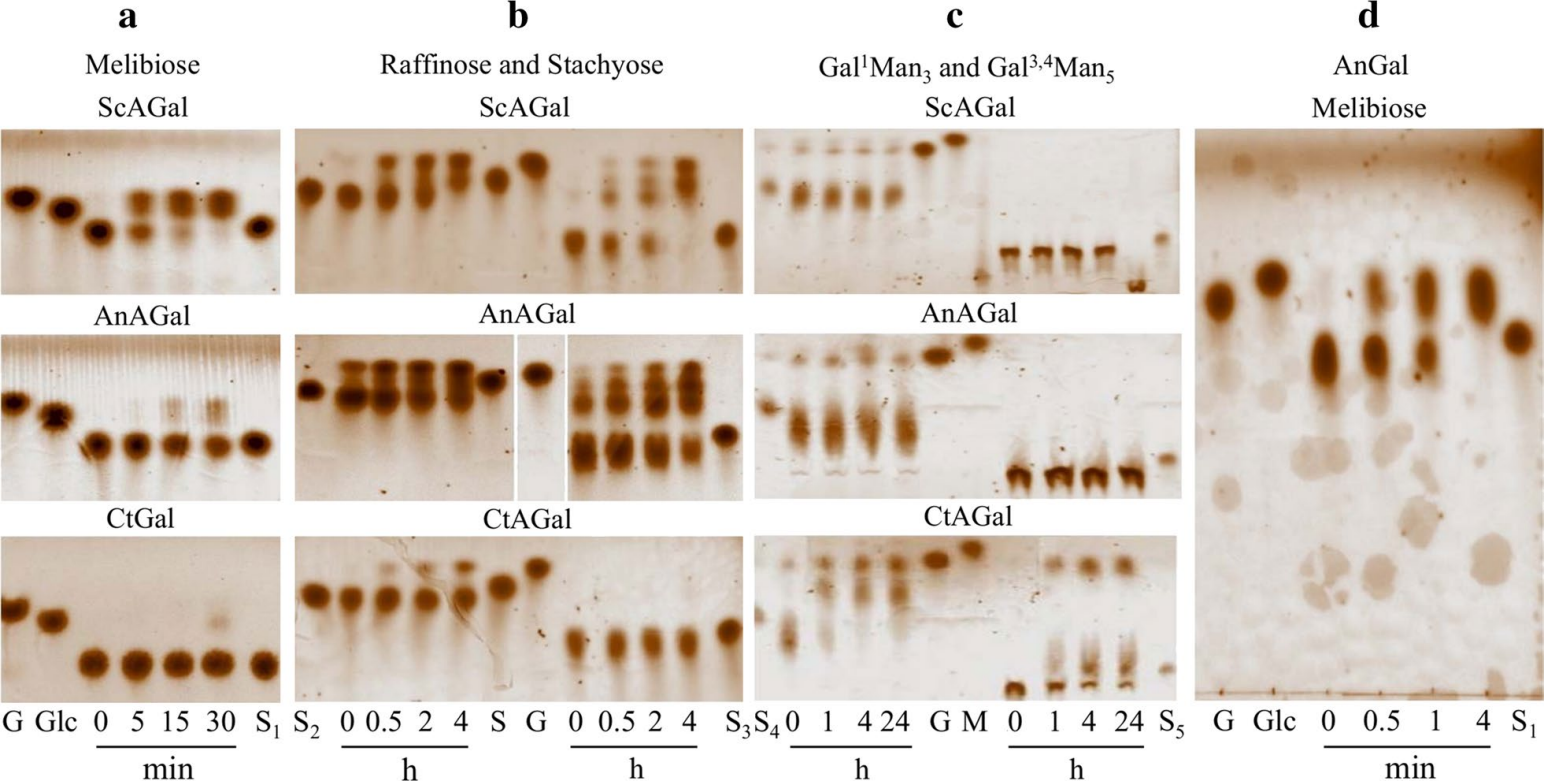
TLC analysis comparing the hydrolytic products by ScAGal, *A. niger* and *C. tetragonoloba* α-Gals. AnAGal, *A. niger* α-Gal; CtAGal, *C. tetragonoloba* α-Gal; S_1_, Melibiose; S_2_, Raffinose; S_3_, Stachyose; S_4_, Gal^1^Man_3_; S_5_, Gal^3,4^Man_5_; G, Galactose; Glc, Glucose; S, Sucrose; M, Mannose

*Comparative study of the hydrolysis of substrates by ScAGal and the α*-*Gals of A. niger and C. tetragonoloba.* There are many α-Gals of different origins and substrate specificity; thus we located those available on the market and compared them with ScAGal. The most accessible were α-Gal of *A. niger* (AnAGal), whose supplier does not provide data about which of the 3 α-Gals characterized hitherto is [67], and α-Gal of *C. tetragonoloba* (CtAGal), which acts on the guar galactomannan [68]. TLC analysis showed that only ScAGal hydrolyses completely melibiose to glucose and galactose after 30 min (Fig. 7a). AnAGal does not act on melibiose even after 12 h [58], but this sugar was totally hydrolysed after 4 h (Fig. 7d). ScAGal has greater specificity for raffinose and stachyose than AnAGal, whereas CtAGal does not act on stachyose and very little on raffinose (Fig. 7b). In contrast, ScAGal and AnAGal cannot hydrolyse Gal^1^Man_3_ and Gal^3,4^Man_5_, whereas CtAGal can hydrolyse these substrates within 1 h (Fig. 7c). These results show that AnAGal as used in these tests could be the AglC, characterized as a tetrameric enzyme that cannot act directly on galactomannans and is classified in the GH36 family. Two other α-Gals, AglA and AglB belonging to the GH27 family, act to a lesser or greater extent on the degradation of galactomannans [67, 69]. Substrate specificity seems to be determined more by molecular state of the protein than similarities in their amino acid sequence. Monomeric enzymes belonging to different families of GH can act on small oligosaccharides and polymeric galactomannans [35, 70, 71]; however, those that are organized as high MW multimeric complexes only can hydrolyse small oligosaccharides [28, 72]. ScAGal is a tetrameric enzyme and is a further example that the inability to act on polymeric substrates might be due to its multimeric structure restricting access to the active site of the enzyme. Additional file 7: Table S4 summarizes the specific substrates of ScAGal, AnAGal and CtAGal.

### Optimization of ScAGal production by RSM

**Fig. 8:**
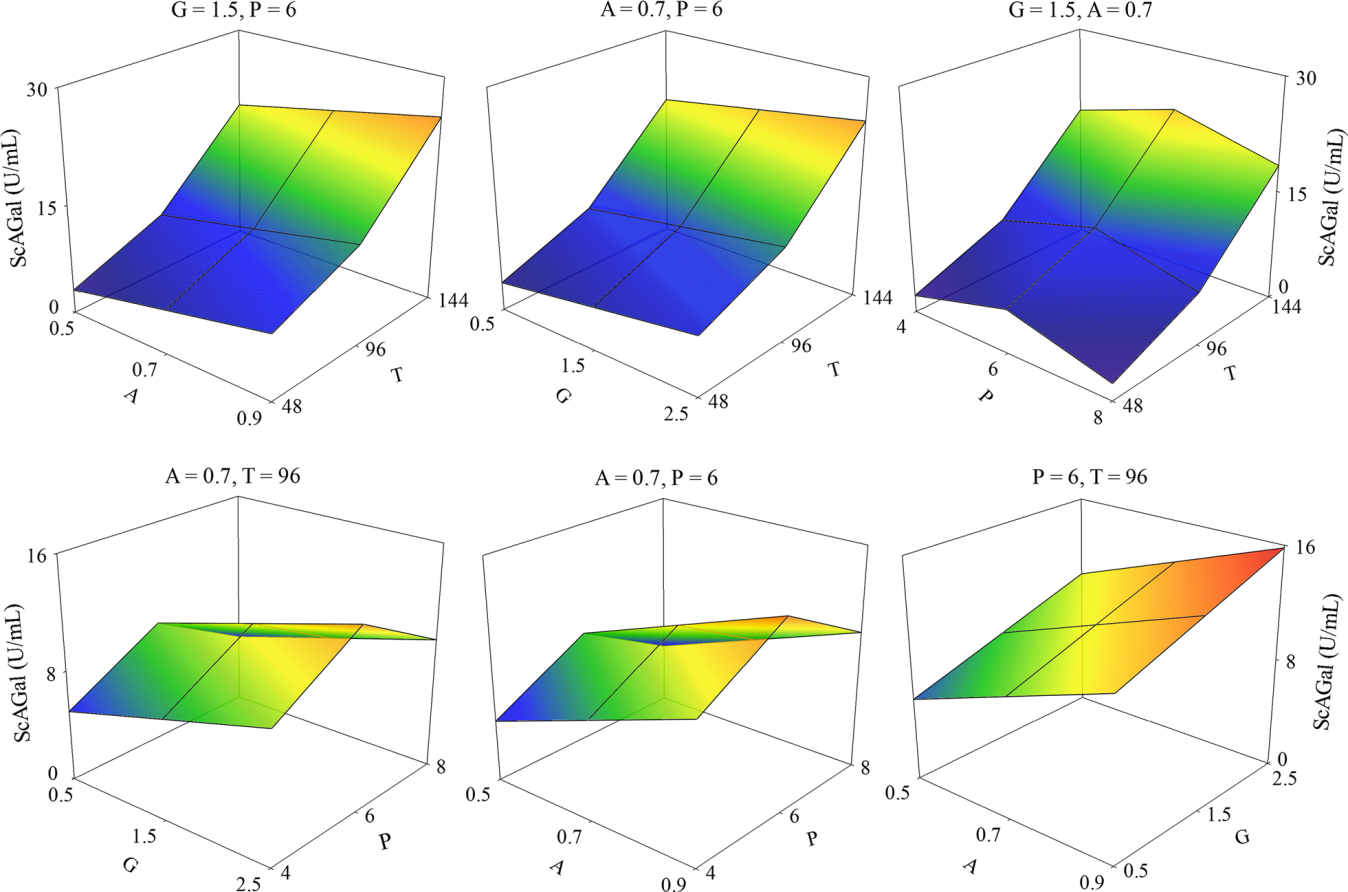
Response surface plots optimizing the production of ScAGal using the data generated in the Additional file 8: Table S5. A, aeration (1 − (Vc/Ve), see Table 1); G, glucose concentration (%); P, pH; T, culture time (h)

**Fig. 9:**
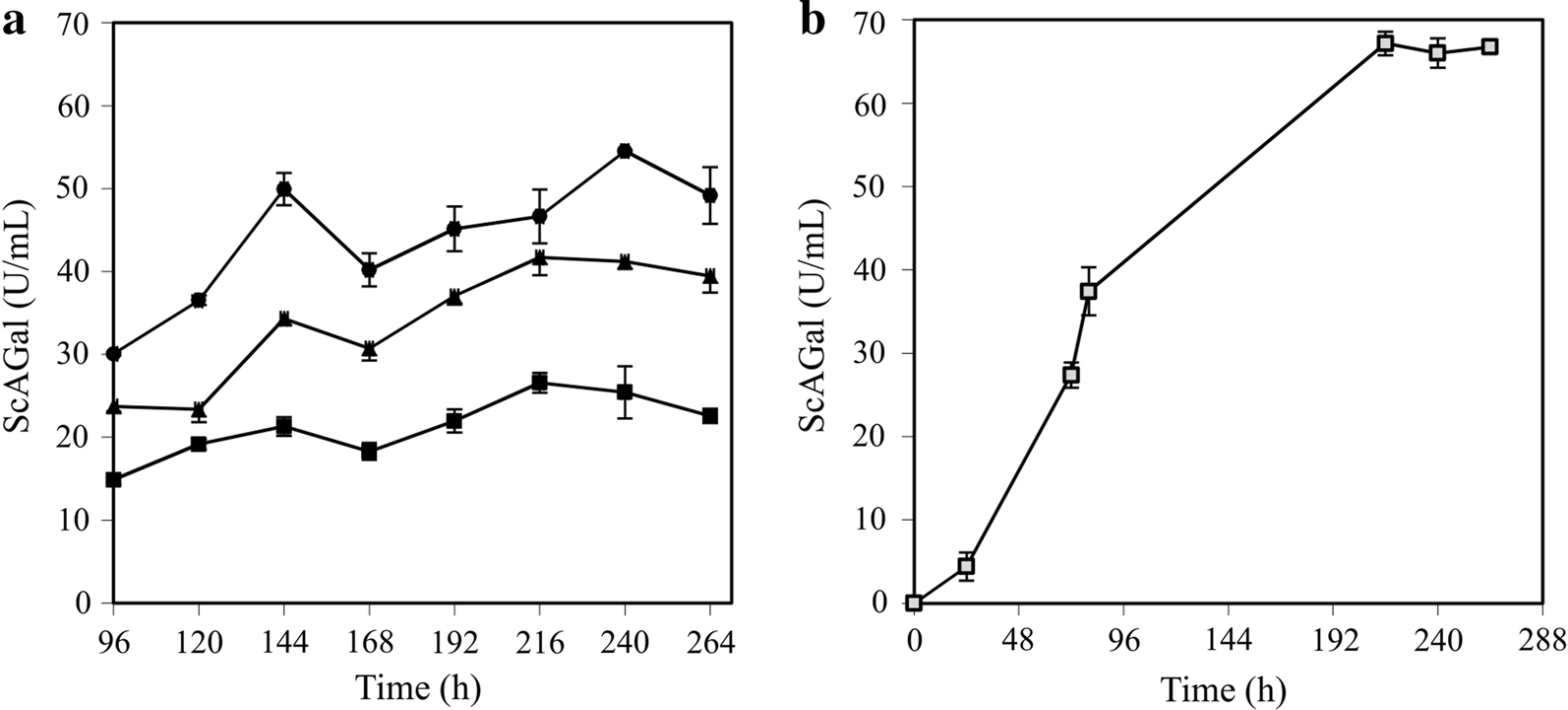
**a** Monitoring of the extracellular α-galactosidase activity of cultures with the recombinant strains BJ3505/YEp*MEL1* (squares), BJ3505/YEp*MEL1*Flag (circles) and BJ3505/YEp*MEL1*His (triangles), using the conditions estimated in the model (A = 0.8, G = 2, P = 6). **b** Maximum extracellular α-galactosidase activity of a culture of BJ3505/YEp*MEL1*His, using an OD_600_ of 10 as preinoculum and keeping the rest of the experimental factors estimated by the model (A = 0.8, G = 1.5, P = 6)

RSM can determine the optimal values of the chosen experimental factors (aeration, glucose concentration, pH and culture time) that assist in reaching the maximum extracellular α-galactosidase activity (response), and therefore improve the production of ScAGal. The CCD matrix led to a set of 30 experiments (4 replicates, point = 0), where coded and real values, and corresponding results adjusted by the RSM, are given in Additional file 8: Table S5. The highest activity (21.09 U/mL) was achieved with 0.7 (30/100) aeration, 1.5% glucose and at pH 6 at 144 h of culture (Experiment 24, Additional file 8: Table S5), whereas it decreased drastically (3.45 U/mL) at 48 h under the same conditions (Experiment 23, Additional file 8: Table S5). ANOVA of the statistical significance of the regression model after the elimination of statistically showed no significant effect (*p*-value > 0.05; Additional file 9: Table S6). The model had been fitted properly to the observed data (lack of fit test, *p*-value > 0.05) and showed a correlation coefficient (*R*^*2*^) that explains 86% of the variability in activity (the remaining 14% being attributed to deviations from the model and not to experimental factors). The regression equation of the adjusted model (extracellular α-galactosidase activity = − 38.7343 + 13.8846A + 2.1334G + 11.1035P − 0.0970T − 0.9252P^2^ + 0.0014T^2^) establishes a significant cause-effect relationship between aeration, glucose concentration and culture time, with that of ScAGal production. Optimal response within the experimental domain was achieved with the conditions: A = + 2 (0.9), G = + 2 (2.5%), P = 0 (pH 6), T = + 2 (144 h). Response surface plots showed the behaviour of the experimental variables and that the culture time is the highest positive effect on the production of ScAGal compared to aeration and glucose concentration (Fig. 8). In this way, we could apply the method of maximum slope in the ascent to estimate the trajectory of the response from the centre of the experimental design (0, 0, 0, 0) generated by the change of the variable T (increments of 24). Moreover, the initial optimal conditions estimated in this work are far from the real optimum because YEp*MEL1*His is subject to catabolic repression and thus, as we increase glucose, the time needed to produce ScAGal increases. Therefore, we recommend a control of glucose concentration to avoid long growth times, and ScAGal should therefore increase in correlation. Figure 9a shows the time-course of the culture of the recombinant strain BJ3505/YEp*MEL1*His, using the estimated conditions from the path of slope, outside the experimental domain; A = 0.8, G = 2, P = 6. A mean of 24 U/mL was detected with 48% increase in activity compared to that previously observed (“Monitoring the expression of variants of MEL1” section, Fig. 2). Similar results were observed in cultures transformed with YEp*MEL1* (52% increase) and YEp*MEL1*Flag (56% increase) under the same conditions (Fig. 9a). In fact, catabolic repression means that the cellular machinery directs its energy expenditure to cell growth before initiating expression of recombinant protein, lengthening the maximum production at 216 h of culture. To avoid this elongation, and since the overexpressed protein is not toxic to the cell, we decided to use a higher cell density (OD_600_ of 10 instead of 0.5) and YPHSM medium with 1.5% glucose (maintaining the rest of the predicted parameters; A = 0.8 and P = 6). This resulted in an activity of 66 U/mL being achieved, and established the maximum production at 190 h of culture (Fig. 9b).

Therefore, optimization of the culture conditions helped to improve the productivity of ScAGal tenfold by increasing the concentration of the carbon source and aeration over a 96 h cultivation period, and up to 20 times by lengthening the growth time to 216 h.

## Conclusion

Different constructions were designed to express α-galactosidase of *Saccharomyces cerevisiae*. Presence of the native secretion signal of the protein allowed the recombinant strain to secrete more α-galactosidase than using the α-Factor secretion signal. This enzyme shows strong resistance to acid-neutral proteases during incubations of up to 16 h, and only acts on complex galactomannans after prior hydrolysis of the substrates with β-mannosidase. The enzyme also belongs to Group 1 of α-galactosidases according to its action on synthetic galactomannan-oligosaccharides, as indicated by TLC and HPLC analysis. Finally, statistical application of the response surface methodology helped to optimize enzyme production in reaching 66 U/mL at 190 h culture in the final conditions that were established (aeration = 0.8, 1.5% glucose, OD_600_ of 10, pH 6).

## Supplementary information

**Additional file 1: Table S1.** Oligonucleotides used in this study.

**Additional file 2: Table S2**. ANOVA of ScAGal expression systems, using YPHSM as the culture medium.

**Additional file 3: Fig. S1.** Plasmid stability (A) and typical profile of cell growth (circle), extracellular (square) and intracellular (triangle) α-galactosidase (B) activity of cultures of BJ3505/YEp*MEL1*His. Mean ± DS, N = 3.

**Additional file 4: Fig. S2.** PAGE analysis of ScAGal. (A) Purification steps in 10% SDS-PAGE: extracellular culture medium (lane 1), concentrated medium (lane 2), protein purified by affinity chromatography (lane 3); (B) Monomeric form in 8% SDS-PAGE: glycosylated and deglycosylated protein (lanes 1 and 2, respectively), deglycosylated protein purified by molecular exclusion (lane 3), freeze-dried deglycosylated protein (lane 4); (C) tetrameric form in 8% Native-PAGE: glycosylated (lane 1) and deglycosylated (lane 2) protein. MW, molecular weight marker.

**Additional file 5: Fig. S3.** Conservation conditions of ScAGal. Residual activity reached from partially purified ScAGal batches stored at −  20 °C and pure ScAGal batches stored at RT (22 ± 2 °C), 4 °C and − 20 °C. (Mean ± DS, N = 3).

**Additional file 6: Table S3.** Substrate specificity of ScAGal (Mean ± DS, N = 3).

**Additional file 7: Table S4.** Substrate specificities of ScAGal and *A. niger* and C. *tetragonoloba* α-Gals.

**Additional file 8: Table S5.** Experimental matrix according to the CCD and results observed and estimated by RSM to optimization of ScAGal production by BJ3505/YEp*MEL1*His.

**Additional file 9: Table S6.** ANOVA for the response surface quadratic model to optimization of ScAGal production by BJ3505/YEp*MEL1*His.

## Data Availability

All the data generated or analysed during this study are included in this published article and its additional files.
