# Supplementary material for: Optimization of Saccharomyces cerevisiae α-galactosidase production and application in the degradation of raffinose family oligosaccharides
Source: Microb Cell Fact. 2019 Oct 10;18:172. doi: 10.1186/s12934-019-1222-x (PMC6786279; doi:10.1186/s12934-019-1222-x)
Supplement: Supplementary file 1 — Additional file 1: Table S1. Oligonucleotides used in this study. [file 12934_2019_1222_MOESM1_ESM.docx]

Additional file 1

Optimization of *Saccharomyces cerevisiae* α-galactosidase production and application in the degradation of raffinose family oligosaccharides

María-Efigenia Álvarez-Cao, María-Esperanza Cerdán, María-Isabel González-Siso and Manuel Becerra*

Universidade da Coruña. Grupo EXPRELA, Centro de Investigacións Científicas Avanzadas (CICA), Departamento de Bioloxía, Facultade de Ciencias, A Coruña, Spain

*Corresponding author‘s e-mail: manu@udc.es

**Table S1.** Oligonucleotides used in this study.

| Sequence (5´ to 3´) ^a^ | Strand ^b^ | Applied strategy |
| --- | --- | --- |
| ccagcattgctgctaaagaagaaggggtacctttggat**aaaaga**GTGTCTCCGAGTTACAATGGC | F | YE*p*αF*MEL1*  YEpαF*MEL1*His  YEpαF*MEL1*Flag |
| cccgggagatctatcgatggatccgggcccGTGTCTCCGAGTTACAATGGC | F | YEpFlag*MEL1* |
| CACTCGAAATACTCTTACTACTGC | F | Inserts sequencing |
| tgggacgctcgacggatcagcggccgcttaTCAGTGGTGGTGGTGGTGGTGAGAAGAGGGTCTCAACCTATA | R | YEpαF*MEL1*His |
| tgggacgctcgacggatcagcggccgcttaTCACTTGTCATCGTCATCCTTGTAGTCAGAAGAGGGTCTCAACCTATA | R | YEpαF*MEL1*Flag |
| tgggacgctcgacggatcagcggccgcttaTCAAGAAGAGGGTCTCAACCT | R | YEpαF*MEL1* YEpFlag*MEL1* |
| GTGTACGCATGTAACATTATACTG | R | Inserts sequencing |

^a^ Engineered sequences contain a recombination zone in YEpFLAG-1 (lowercase) and a zone homologous to the target gene (upper case). In bold, the coding sequence of the Kex endoprotease. Purification labels (Poly-His peptide, Flag peptide) are underlined. ^b^ F, forward strand; R, reverse strand.
