# Supplementary material for: Optimization of Saccharomyces cerevisiae α-galactosidase production and application in the degradation of raffinose family oligosaccharides
Source: Microb Cell Fact. 2019 Oct 10;18:172. doi: 10.1186/s12934-019-1222-x (PMC6786279; doi:10.1186/s12934-019-1222-x)
Supplement: Supplementary file 2 — Additional file 2: Table S2. ANOVA of ScAGal expression systems, using YPHSM as the culture medium. [file 12934_2019_1222_MOESM2_ESM.docx]

Additional file 2

Optimization of *Saccharomyces cerevisiae* α-galactosidase production and application in the degradation of raffinose family oligosaccharides

María-Efigenia Álvarez-Cao, María-Esperanza Cerdán, María-Isabel González-Siso and Manuel Becerra*

Universidade da Coruña. Grupo EXPRELA, Centro de Investigacións Científicas Avanzadas (CICA), Departamento de Bioloxía, Facultade de Ciencias, A Coruña, Spain

*Corresponding author‘s e-mail: manu@udc.es

**Table S2.** ANOVA of ScAGal expression systems, using YPHSM as the culture medium.

| STATISTICAL SUMMARY | | | | | | |
| --- | --- | --- | --- | --- | --- | --- |
| Expression systems (Groups) | | Mean | Minimum | Maximum | Bias | Curtosis |
| BJ3505/YEp*MEL1* (A) | | 21.049 | 14.571 | 26.209 | -0.644 | 0.685 |
| BJ3505/YEp*MEL1*Flag (B) | | 23.329 | 15.604 | 26.560 | -1.525 | 1.429 |
| BJ3505/YEp*MEL1*His (C) | | 9.886 | 5.434 | 12.418 | -1.274 | 1.067 |
| BJ3505/YEpFlag*MEL1* (D) | | 1.274 | 0.624 | 1.721 | -0.759 | -0.099 |
| BJ3505/YEpαF*MEL1* (E) | | 2.244 | 0.965 | 3.065 | -0.564 | -0.877 |
| BJ3505/YEpαF*MEL1*Flag (F) | | 2.772 | 1.412 | 3.790 | -0.504 | -0.765 |
| BJ3505/YEpαF*MEL1*His (G) | | 1.403 | 0.614 | 2.135 | -0.137 | -1.117 |
| ANOVA ^a^ | | | | | | |
| Source of variation | | SS | DF | MS | F*-*value | *p*-value |
| Between-Groups | | 414.066 | 2 | 207.033 | 10.36 | 0.0046 |
| Within-Groups | | 179.930 | 9 | 19.992 |  |  |
| Total (Corrected) | | 593.996 | 11 |  |  |  |
| MULTIPLE RANGE TEST ^b^ | | | | | | |
| Contrast | Significant ^c^ | Different | +/- Limits |  |  |  |
| A – B |  | -2.28 | 7.15219 |  |  |  |
| A – C | * | 11.1635 | 7.15219 |  |  |  |
| B – C | * | 13.4435 | 7.15219 |  |  |  |

^a^ Response (independent variable) is the extracellular α-galactosidase activity of the cultures tested. ^b^ Fisher's Least Significant Difference test was used to discriminate between the means. ^c^ Denotes a statistically significant difference between pairs of experimental groups (contrast). SS, sum of square; DF, degrees of freedom; MS, mean square.
