# Supplementary material for: Optimization of Saccharomyces cerevisiae α-galactosidase production and application in the degradation of raffinose family oligosaccharides
Source: Microb Cell Fact. 2019 Oct 10;18:172. doi: 10.1186/s12934-019-1222-x (PMC6786279; doi:10.1186/s12934-019-1222-x)
Supplement: Supplementary file 3 — Additional file 3: Fig. S1. Plasmid stability (A) and typical profile of cell growth (circle), extracellular (square) and intracellular (triangle) α-galactosidase (B) activity of cultures of BJ3505/YEpMEL1His. Mean ± DS, N = 3. [file 12934_2019_1222_MOESM3_ESM.docx]

Additional file 3

Optimization of *Saccharomyces cerevisiae* α-galactosidase production and application in the degradation of raffinose family oligosaccharides

María-Efigenia Álvarez-Cao, María-Esperanza Cerdán, María-Isabel González-Siso and Manuel Becerra*

Universidade da Coruña. Grupo EXPRELA, Centro de Investigacións Científicas Avanzadas (CICA), Departamento de Bioloxía, Facultade de Ciencias, A Coruña, Spain

*Corresponding author‘s e-mail: manu@udc.es

**
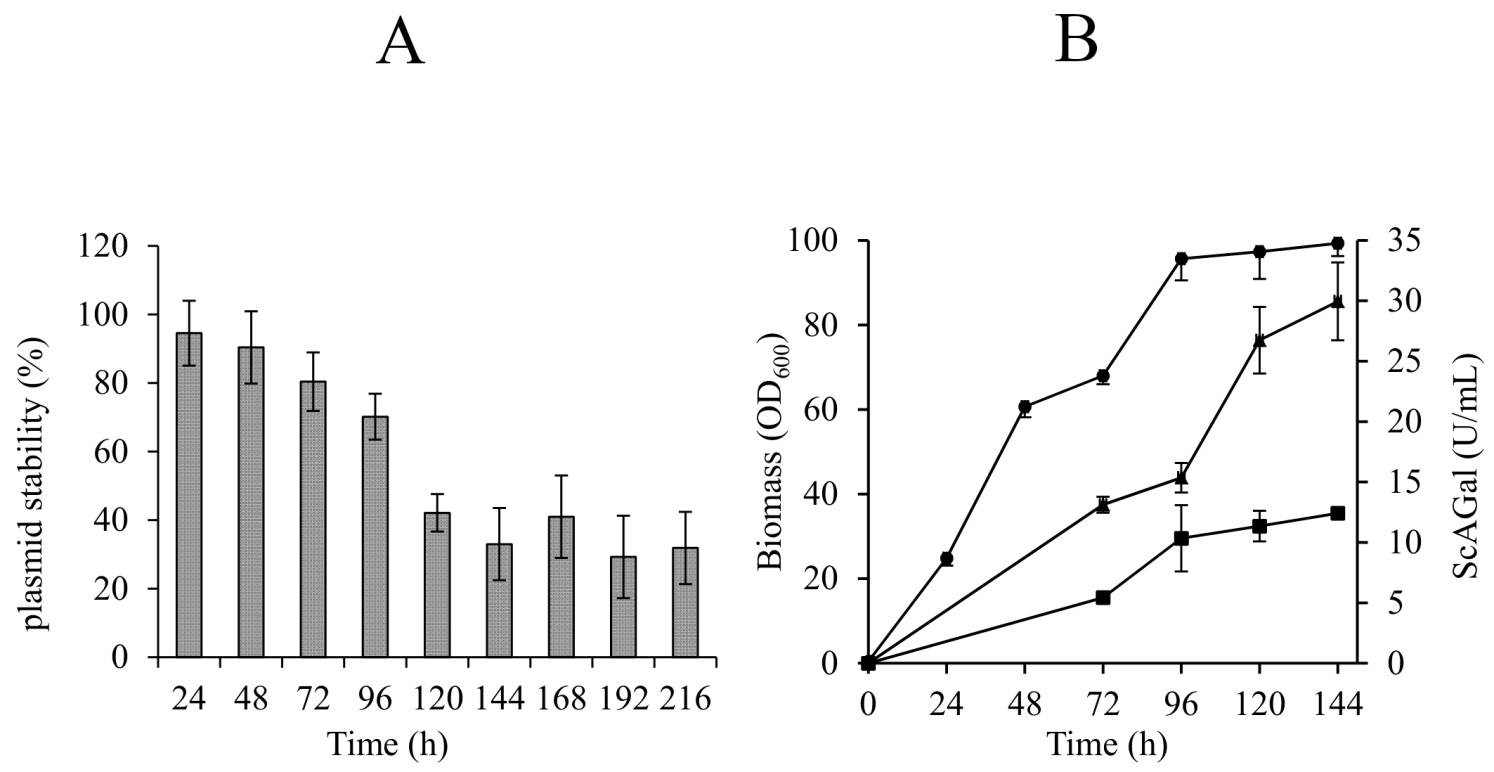
**

**Fig. S1.** Plasmid stability (A) and typical profile of cell growth (circle), extracellular (square) and intracellular (triangle) α-galactosidase (B) activity of cultures of BJ3505/YEp*MEL1*His. Mean ± DS, N = 3.
