# Supplementary material for: Optimization of Saccharomyces cerevisiae α-galactosidase production and application in the degradation of raffinose family oligosaccharides
Source: Microb Cell Fact. 2019 Oct 10;18:172. doi: 10.1186/s12934-019-1222-x (PMC6786279; doi:10.1186/s12934-019-1222-x)
Supplement: Supplementary file 4 — Additional file 4: Fig. S2. PAGE analysis of ScAGal. (A) Purification steps in 10% SDS-PAGE: extracellular culture medium (lane 1), concentrated medium (lane 2), protein purified by affinity chromatography (lane 3); (B) Monomeric form in 8% SDS-PAGE: glycosylated and deglycosylated protein (lanes 1 and 2, respectively), deglycosylated protein purified by molecular exclusion (lane 3), freeze-dried deglycosylated protein (lane 4); (C) tetrameric form in 8% Native-PAGE: glycosylated (lane 1) and deglycosylated (lane 2) protein. MW, molecular weight marker. [file 12934_2019_1222_MOESM4_ESM.docx]

Additional file 4

Optimization of *Saccharomyces cerevisiae* α-galactosidase production and application in the degradation of raffinose family oligosaccharides

María-Efigenia Álvarez-Cao, María-Esperanza Cerdán, María-Isabel González-Siso and Manuel Becerra*

Universidade da Coruña. Grupo EXPRELA, Centro de Investigacións Científicas Avanzadas (CICA), Departamento de Bioloxía, Facultade de Ciencias, A Coruña, Spain

*Corresponding author‘s e-mail: manu@udc.es


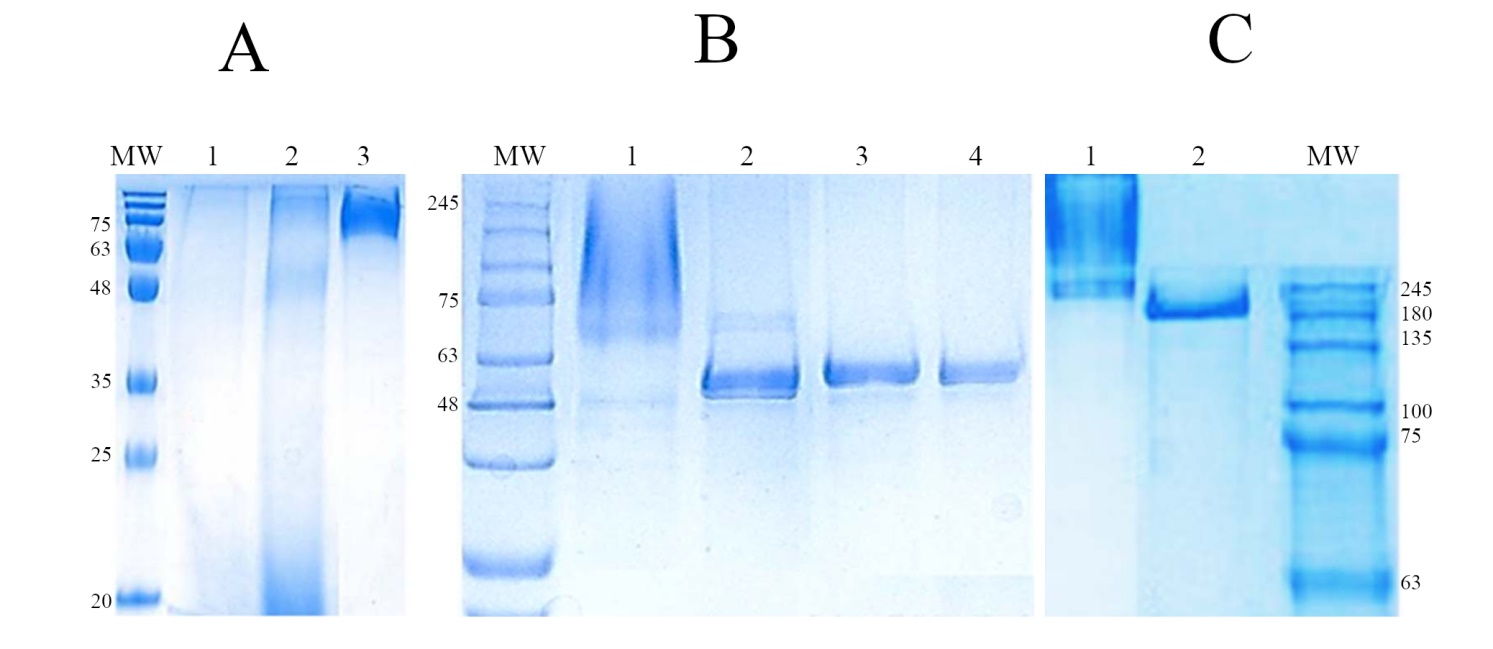


**Fig. S2.** PAGE analysis of ScAGal. (A) Purification steps in 10% SDS-PAGE: 28 μg of extracellular culture medium (lane 1), 16 μg of concentrated medium (lane 2), 3.5 μg of protein purified by affinity chromatography (lane 3); (B) Monomeric form in 8% SDS-PAGE: glycosylated (7 μg) and deglycosylated (2 μg) protein (lanes 1 and 2, respectively), 1.8 μg of deglycosylated protein purified by molecular exclusion (lane 3), 0.5 μg of freeze-dried deglycosylated protein (lane 4); (C) Tetrameric form in 8% Native-PAGE: 7 μg of glycosylated (lane 1) and 2 μg of deglycosylated (lane 2) protein. MW, molecular weight marker (NZYColour Protein Marker II, Nzytech).
