# Supplementary material for: Optimization of Saccharomyces cerevisiae α-galactosidase production and application in the degradation of raffinose family oligosaccharides
Source: Microb Cell Fact. 2019 Oct 10;18:172. doi: 10.1186/s12934-019-1222-x (PMC6786279; doi:10.1186/s12934-019-1222-x)
Supplement: Supplementary file 5 — Additional file 5: Fig. S3. Conservation conditions of ScAGal. Residual activity reached from partially purified ScAGal batches stored at − 20 °C and pure ScAGal batches stored at RT (22 ± 2 °C), 4 °C and − 20 °C. (Mean ± DS, N = 3). [file 12934_2019_1222_MOESM5_ESM.docx]

Additional file 5

Optimization of *Saccharomyces cerevisiae* α-galactosidase production and application in the degradation of raffinose family oligosaccharides

María-Efigenia Álvarez-Cao, María-Esperanza Cerdán, María-Isabel González-Siso and Manuel Becerra*

Universidade da Coruña. Grupo EXPRELA, Centro de Investigacións Científicas Avanzadas (CICA), Departamento de Bioloxía, Facultade de Ciencias, A Coruña, Spain

*Corresponding author‘s e-mail: manu@udc.es


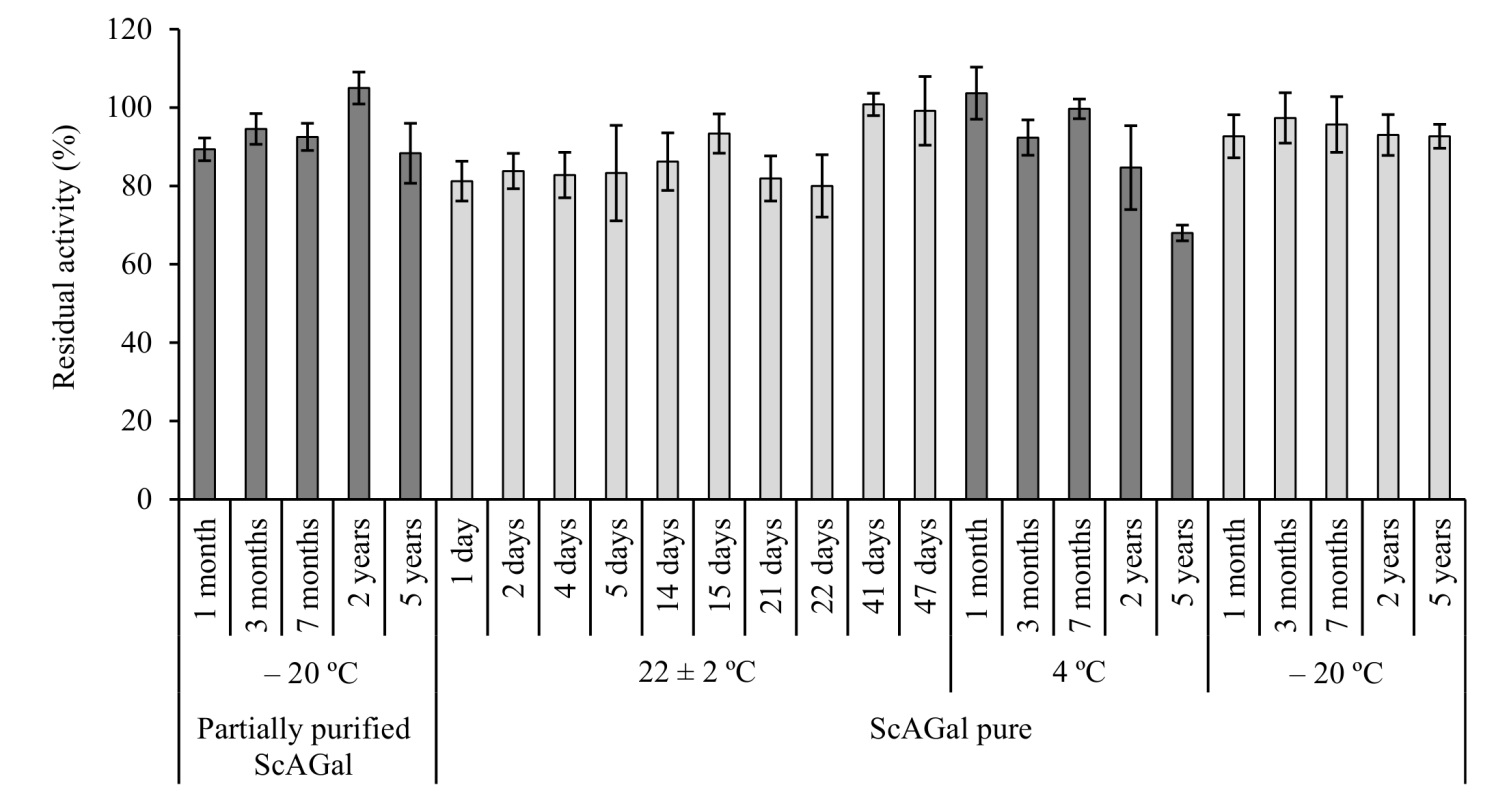


**Fig. S3.** Conservation conditions of ScAGal. Residual activity reached from partially purified ScAGal batches stored at -20°C and pure ScAGal batches stored at RT (22 ± 2°C), 4°C and -20°C. (Mean ± DS, N = 3).
