# Supplementary material for: Optimization of Saccharomyces cerevisiae α-galactosidase production and application in the degradation of raffinose family oligosaccharides
Source: Microb Cell Fact. 2019 Oct 10;18:172. doi: 10.1186/s12934-019-1222-x (PMC6786279; doi:10.1186/s12934-019-1222-x)
Supplement: Supplementary file 6 — Additional file 6: Table S3. Substrate specificity of ScAGal (Mean ± DS, N = 3). [file 12934_2019_1222_MOESM6_ESM.docx]

Additional file 6

Optimization of *Saccharomyces cerevisiae* α-galactosidase production and application in the degradation of raffinose family oligosaccharides

María-Efigenia Álvarez-Cao, María-Esperanza Cerdán, María-Isabel González-Siso and Manuel Becerra*

Universidade da Coruña. Grupo EXPRELA, Centro de Investigacións Científicas Avanzadas (CICA), Departamento de Bioloxía, Facultade de Ciencias, A Coruña, Spain

*Corresponding author‘s e-mail: manu@udc.es

**Table S3.** Substrate Specificity of ScAGal (Mean ± DS, N = 3).

| Substrate | Concentration (mg/ml) | Relative activity ^a^ (%) |
| --- | --- | --- |
| PNPG | 3 | 100± 0.063 |
| Melibiose | 5 | 95 ± 0.11 |
| Raffinose | 5 | 15 ± 0.01 |
| Stachyose | 5 | 11 ± 0.01 |
| Locust bean gum | 5 | nd ^b^ |
| Locust bean gum + β-manosidasa | 2.5 | 1 ± 0.00 |

^a^ Relative activities were calculated in relation to PNPG activity, which was considered as 100%. ^b^ Not detected.
