# Supplementary material for: Optimization of Saccharomyces cerevisiae α-galactosidase production and application in the degradation of raffinose family oligosaccharides
Source: Microb Cell Fact. 2019 Oct 10;18:172. doi: 10.1186/s12934-019-1222-x (PMC6786279; doi:10.1186/s12934-019-1222-x)
Supplement: Supplementary file 7 — Additional file 7: Table S4. Substrate specificities of ScAGal and A. niger and C. tetragonoloba α-Gals. [file 12934_2019_1222_MOESM7_ESM.docx]

Additional file 7

Optimization of *Saccharomyces cerevisiae* α-galactosidase production and application in the degradation of raffinose family oligosaccharides

María-Efigenia Álvarez-Cao, María-Esperanza Cerdán, María-Isabel González-Siso and Manuel Becerra*

Universidade da Coruña. Grupo EXPRELA, Centro de Investigacións Científicas Avanzadas (CICA), Departamento de Bioloxía, Facultade de Ciencias, A Coruña, Spain

*Corresponding author‘s e-mail: manu@udc.es

**Table S4.** Substrate specificities of ScAGal and *A. niger* and *C. tetragonoloba* α-Gals

| Substrate | ScAGal | AnGal | CtAGal |
| --- | --- | --- | --- |
| Melibiose | Gal–Glc | Gal–Glc | Gal–Glc |
|  | **⮅** | ⭱ | ⭱ |
| Raffinose | Gal–Glc–Fru | Gal–Glc–Fru | Gal–Glc–Fru |
|  | **⮅** | **⭡** | ⭫ |
| Stachyose | Gal–Gal–Glc–Fru | Gal–Gal–Glc–Fru | Gal–Gal–Glc–Fru |
|  | **⮅ ⮅** | **⭡ ⭡** | ⭱ ⭱ |
| Gal^1^Man_3_ | Gal | Gal | Gal |
|  | **⭲│** | **⭲│** | **⮆│** |
|  | M^b^–M–M | M^b^–M–M | M^b^–M–M |
|  | ⇧ ⇧ |  |  |
| GalMan^a^ | M–Gal | na^c^ | na^c^ |
|  | **⭡** |  |  |
| Gal^3,4^Man_5_ | Gal Gal | Gal Gal | Gal Gal |
|  | **⭲│ ⭲│** | **⭲│⭲│** | **⮆│⮆│** |
|  | M^b^–M–M–M–M | M^b^–M–M–M–M | M^b^–M–M–M–M |
|  | ⇧ |  |  |
| Gal^3,4^Man_4_^a^ | Gal Gal | na^c^ | na^c^ |
|  | **⭬│⭲│** |  |  |
|  | M–M–M–M |  |  |

^a^ Substrates obtained by β-mannosidase. ^b^ Non-reducing mannose.^c^ Not assayed. AnGal, *A. niger* α-Gal; CtAGal, *C. tetragonoloba* α-Gal; Gal, galactose residue; Glc, glucose residue; Fru, fructose residue; M, mannose residue; **⮅**, linkages rapidly hydrolyzed by α-Gal; **⭡**, linkages moderately hydrolyzed by α-Gal; ⭫, linkages weakly hydrolyzed by α-Gal; **⭲**, linkages hardly or not hydrolyzed by α-Gal; ⇧, linkages hydrolyzed by β-mannosidase.
