# Supplementary material for: Optimization of Saccharomyces cerevisiae α-galactosidase production and application in the degradation of raffinose family oligosaccharides
Source: Microb Cell Fact. 2019 Oct 10;18:172. doi: 10.1186/s12934-019-1222-x (PMC6786279; doi:10.1186/s12934-019-1222-x)
Supplement: Supplementary file 8 — Additional file 8: Table S5. Experimental matrix according to the CCD and results observed and estimated by RSM to optimization of ScAGal production by BJ3505/YEpMEL1His. [file 12934_2019_1222_MOESM8_ESM.docx]

Additional file 8

Optimization of *Saccharomyces cerevisiae* α-galactosidase production and application in the degradation of raffinose family oligosaccharides

María-Efigenia Álvarez-Cao, María-Esperanza Cerdán, María-Isabel González-Siso and Manuel Becerra*

Universidade da Coruña. Grupo EXPRELA, Centro de Investigacións Científicas Avanzadas (CICA), Departamento de Bioloxía, Facultade de Ciencias, A Coruña, Spain

*Corresponding author‘s e-mail: manu@udc.es

**Table S5.** Experimental matrix according to the CCD and results observed and estimated by RSM to optimization of ScAGal production by BJ3505/YEp*MEL1*His.

| Exp  no. | Coded values | | | |  | Real values ^a^ | | | |  | ScAGal (U/mL)^b^ | |
| --- | --- | --- | --- | --- | --- | --- | --- | --- | --- | --- | --- | --- |
|  | *x_1_* | *x_2_* | *x_3_* | *x_4_* |  | X_1_ | X_2_ | X_3_ | X_4_ |  | Observed | Estimated (*Y*) |
| 1 | -1 | -1 | -1 | -1 |  | 0.6 | 1 | 5 | 72 |  | 5.73 | 4.29 |
| 2 | 1 | -1 | -1 | -1 |  | 0.8 | 1 | 5 | 72 |  | 8.86 | 7.07 |
| 3 | -1 | 1 | -1 | -1 |  | 0.6 | 2 | 5 | 72 |  | 10.12 | 6.43 |
| 4 | 1 | 1 | -1 | -1 |  | 0.8 | 2 | 5 | 72 |  | 11.30 | 9.21 |
| 5 | -1 | -1 | 1 | -1 |  | 0.6 | 1 | 7 | 72 |  | 4.90 | 4.29 |
| 6 | 1 | -1 | 1 | -1 |  | 0.8 | 1 | 7 | 72 |  | 6.87 | 7.07 |
| 7 | -1 | 1 | 1 | -1 |  | 0.6 | 2 | 7 | 72 |  | 6.64 | 6.43 |
| 8 | 1 | 1 | 1 | -1 |  | 0.8 | 2 | 7 | 72 |  | 8.40 | 9.21 |
| 9 | -1 | -1 | -1 | 1 |  | 0.6 | 1 | 5 | 120 |  | 13.77 | 1.,37 |
| 10 | 1 | -1 | -1 | 1 |  | 0.8 | 1 | 5 | 120 |  | 17.77 | 15.14 |
| 11 | -1 | 1 | -1 | 1 |  | 0.6 | 2 | 5 | 120 |  | 14.24 | 14.50 |
| 12 | 1 | 1 | -1 | 1 |  | 0.8 | 2 | 5 | 120 |  | 19.59 | 17.28 |
| 13 | -1 | -1 | 1 | 1 |  | 0.6 | 1 | 7 | 120 |  | 11.92 | 12.37 |
| 14 | 1 | -1 | 1 | 1 |  | 0.8 | 1 | 7 | 120 |  | 12.89 | 15.14 |
| 15 | -1 | 1 | 1 | 1 |  | 0.6 | 2 | 7 | 120 |  | 14.47 | 14.50 |
| 16 | 1 | 1 | 1 | 1 |  | 0.8 | 2 | 7 | 120 |  | 19.73 | 17.28 |
| 17 | -2 | 0 | 0 | 0 |  | 0.9 | 1.5 | 6 | 96 |  | 8.34 | 8.14 |
| 18 | 2 | 0 | 0 | 0 |  | 0.5 | 1.5 | 6 | 96 |  | 13.20 | 13.69 |
| 19 | 0 | -2 | 0 | 0 |  | 0.7 | 0.5 | 6 | 96 |  | 7.71 | 8.78 |
| 20 | 0 | 2 | 0 | 0 |  | 0.7 | 2.5 | 6 | 96 |  | 9.62 | 13.05 |
| 21 | 0 | 0 | -2 | 0 |  | 0.7 | 1.5 | 4 | 96 |  | 4.37 | 7.21 |
| 22 | 0 | 0 | 2 | 0 |  | 0.7 | 1.5 | 8 | 96 |  | 6.40 | 7.21 |
| 23 | 0 | 0 | 0 | -2 |  | 0.7 | 1.5 | 6 | 48 |  | 3.45 | 6.02 |
| 24 | 0 | 0 | 0 | 2 |  | 0.7 | 1.5 | 6 | 144 |  | 21.09 | 22.17 |
| 25 | 0 | 0 | 0 | 0 |  | 0.7 | 1.5 | 6 | 96 |  | 9.97 | 10.92 |
| 26 | 0 | 0 | 0 | 0 |  | 0.7 | 1.5 | 6 | 96 |  | 10.20 | 10.92 |
| 27 | 0 | 0 | 0 | 0 |  | 0.7 | 1.5 | 6 | 96 |  | 11.42 | 10.92 |
| 28 | 0 | 0 | 0 | 0 |  | 0.7 | 1.5 | 6 | 96 |  | 10.31 | 10.92 |
| 29 | 0 | 0 | 0 | 0 |  | 0.7 | 1.5 | 6 | 96 |  | 10.18 | 10.92 |
| 30 | 0 | 0 | 0 | 0 |  | 0.7 | 1.5 | 6 | 96 |  | 10.91 | 10.92 |

^a^ X_1_, aeration (1 - (Vc/Ve), see Table 1); X_2_, glucose concentration (%); X_3_, pH; X_4_, culture time (h). To facilitate the understanding of the results, X_1_, X_2_, X_3_, X_4_ were named as A, B, C, D, respectively. ^b^ Extracellular α-galactosidase activity (μmol.min.mL^-1^) to pH 4 and 40 ºC.
