# Supplementary material for: Optimization of Saccharomyces cerevisiae α-galactosidase production and application in the degradation of raffinose family oligosaccharides
Source: Microb Cell Fact. 2019 Oct 10;18:172. doi: 10.1186/s12934-019-1222-x (PMC6786279; doi:10.1186/s12934-019-1222-x)
Supplement: Supplementary file 9 — Additional file 9: Table S6. ANOVA for the response surface quadratic model to optimization of ScAGal production by BJ3505/YEpMEL1His. [file 12934_2019_1222_MOESM9_ESM.docx]

Additional file 9

Optimization of *Saccharomyces cerevisiae* α-galactosidase production and application in the degradation of raffinose family oligosaccharides

María-Efigenia Álvarez-Cao, María-Esperanza Cerdán, María-Isabel González-Siso and Manuel Becerra*

Universidade da Coruña. Grupo EXPRELA, Centro de Investigacións Científicas Avanzadas (CICA), Departamento de Bioloxía, Facultade de Ciencias, A Coruña, Spain

*Corresponding author‘s e-mail: manu@udc.es

**Table S6.** ANOVA for the response surface quadratic model to optimization of ScAGal production by BJ3505/YEp*MEL1*His^a^.

| Effects ^b^ | Sum of squares | Df ^c^ | Mean square | F-ratio | *p*-value ^d^ |
| --- | --- | --- | --- | --- | --- |
| A: Aeration | 46.27 | 1 | 46.27 | 21.72 | 0.0004 |
| B: Glucose | 27.31 | 1 | 27.31 | 12.82 | 0.0030 |
| D: Time | 390.94 | 1 | 390.94 | 183.53 | < 0.0001 |
| CC | 24.35 | 1 | 24.35 | 11.43 | 0.0045 |
| DD | 17.99 | 1 | 17.99 | 8.44 | 0.0115 |
| Lack of Fit | 52.43 | 10 | 5.24 | 2.46 | 0.0603 |
| Pure Error | 29.82 | 14 | 2.13 |  |  |
| Total | 594.34 | 29 |  |  |  |

^a^ *R^2^* = 86.16 %; adjusted *R^2^* = 83.28%; standard error = 1.46; mean absolute error = 1.29. ^b^ Quadratic effects of pH (CC) and culture time (DD). ^c^ Df, Degrees of freedom. ^d^ *p* ≤ 0.05 denotes a statistically significant difference.
